# Decked Out for Success: A Novel Card Game to Support School Teaching of Radioactivity and Nuclear Science

**DOI:** 10.1021/acs.jchemed.4c00603

**Published:** 2024-12-18

**Authors:** Sarah E. Lu, Shaun D. Hemming, Jamie M. Purkis

**Affiliations:** University of Southampton, University Road, Southampton SO17 1BJ, United Kingdom (U.K.)

**Keywords:** Gamification, Nuclear, Radiation, Climate Change, Nuclear Skills Gap

## Abstract

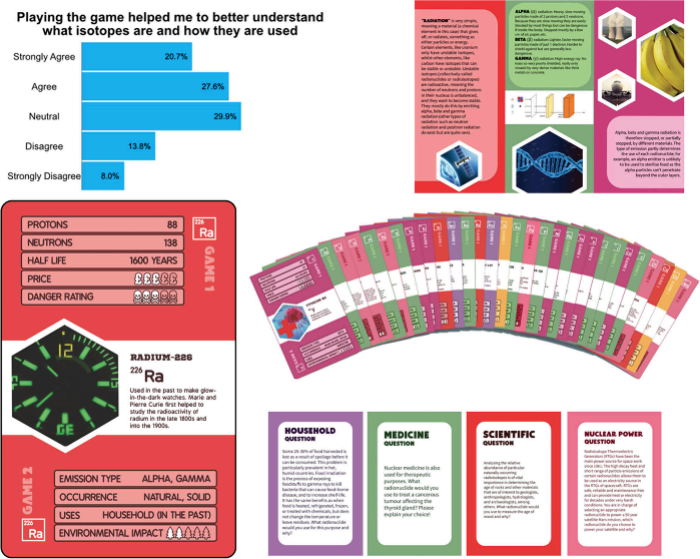

The global nuclear skills shortage requires a comprehensive investment in training at all levels of education. With focus on post-18 and vocational education, there is a lack of resource and awareness for teaching nuclear skills to students between the ages of 11 to 18 years of age. This age group is vital if interest in this industry is to be nurtured and the skills gap is to be addressed. Here, we report an interactive card game *RAD Ratings* to address this gap; a curriculum-enriching activity, teaching nuclear skills to pre-18 years of age. We emphasize curriculum linked and practical examples in everyday life to make it relatable to students. Student and teacher feedback demonstrated that >64% students enjoyed playing *RAD Ratings*, with >50% students saying that they would play the game again and all teachers surveyed stating that *Rad Ratings* improved student understanding of radionuclides and their uses. Our approach stands out as the sole UK study focused on the gamification of nuclear science and radiochemistry education, uniquely evaluating feedback from both students and teachers concurrently.

## Introduction

The nuclear sector will require considerable workforce expansion in coming decades; this not only relates to the decommissioning of current aging infrastructure, but also to satisfy the predicted demand in the growth of nuclear power to achieve “net zero” targets by the middle of this century.^1,2^ Nuclear science and radiochemistry also play critical roles in nuclear medicine, power, and waste management, as well as agriculture and manufacturing.^3−5^

The industrial and economic growth involving nuclear science and technology necessitates an increase in the demand for workforce development in the nuclear sector.^2,3,6,7^ This increase in workforce demand, coupled with the decline of radiochemistry research and teaching,^5,8^ have led to a shortage in the radiochemistry and nuclear workforce. Addressing this skills shortage is a complex problem, requiring skills learned from apprenticeships, undergraduate programs, and doctoral training, as well as experiential learning (“learning on the job”).^6^ Critically, this also involves working with schools to increase the supply of undergraduates choosing to study nuclear-relevant skills or on-the-job training.^7,9^

However, outreach and workshop activities delivered by the public sector, private sector and academic institutions are unable to effectively compensate or disseminate knowledge evenly across a large student body.^10−12^ Moreover, safety concerns regarding radioactivity in the classroom restrict the hands-on activities that can be used to teach nuclear chemistry concepts. Recently, the COVID-19 pandemic has further decreased these outreach activities, reducing student engagement and teaching of nuclear science to students.^10−12^

Gamification, which involves using games as enjoyable activities for teaching, is a promising strategy to enhance engagement and learning in nuclear and radiochemical education, thereby increasing the level of participation among students and teachers. Educational games have been successfully developed and utilized in classroom settings to enhance and supplement topics covering chemical elements and the periodic table.^12−17^ Educational games are known to improve learning outcomes, particularly for the teaching of chemistry.^13−19^ This approach has recently been demonstrated through two examples, focusing on the teaching of radioactivity.^12,20^

### Radioactivity and ^235^U

An educational card game developed by Martin et al.,^12^ (New York, USA) to introduce students to the principles of radioactive decay, shielding, and the uses of radioactive isotopes. Evaluation undergraduate and first year graduate students’ demonstrated a positive shift in perception of radioactivity and nuclear chemistry, with feedback indicating that radioactivity and ^235^U effectively enhances post-18 students’ knowledge.^12^

### Isotope Rummy

An interactive game (“Isotope Rummy”) developed for classes of 12–16 year olds, accessible through the Cornell Center for Materials Research (CCMR),^20^ focusing on the nucleus and radioactive decay. Feedback suggested gamifying these concepts helped teachers convey the basic concepts of nuclear chemistry.^20^

Building on these studies, we have developed a resource specifically for the United Kingdom (UK) educational syllabus for students pre-18 years of age. This paper describes a UK-first gamification resource for the teaching of radioactivity and nuclear science in schools, specifically for teaching in English schools (Education is a devolved matter for the nations of the UK). Together with the accompanying Supporting Information 1, 2, and 3, which contains full details of our bespoke card game, *RAD Ratings*, it is our hope that this information will be of use to teachers and professionals for teaching of nuclear skills in England and throughout the UK.

## Methods

**Figure 1:**
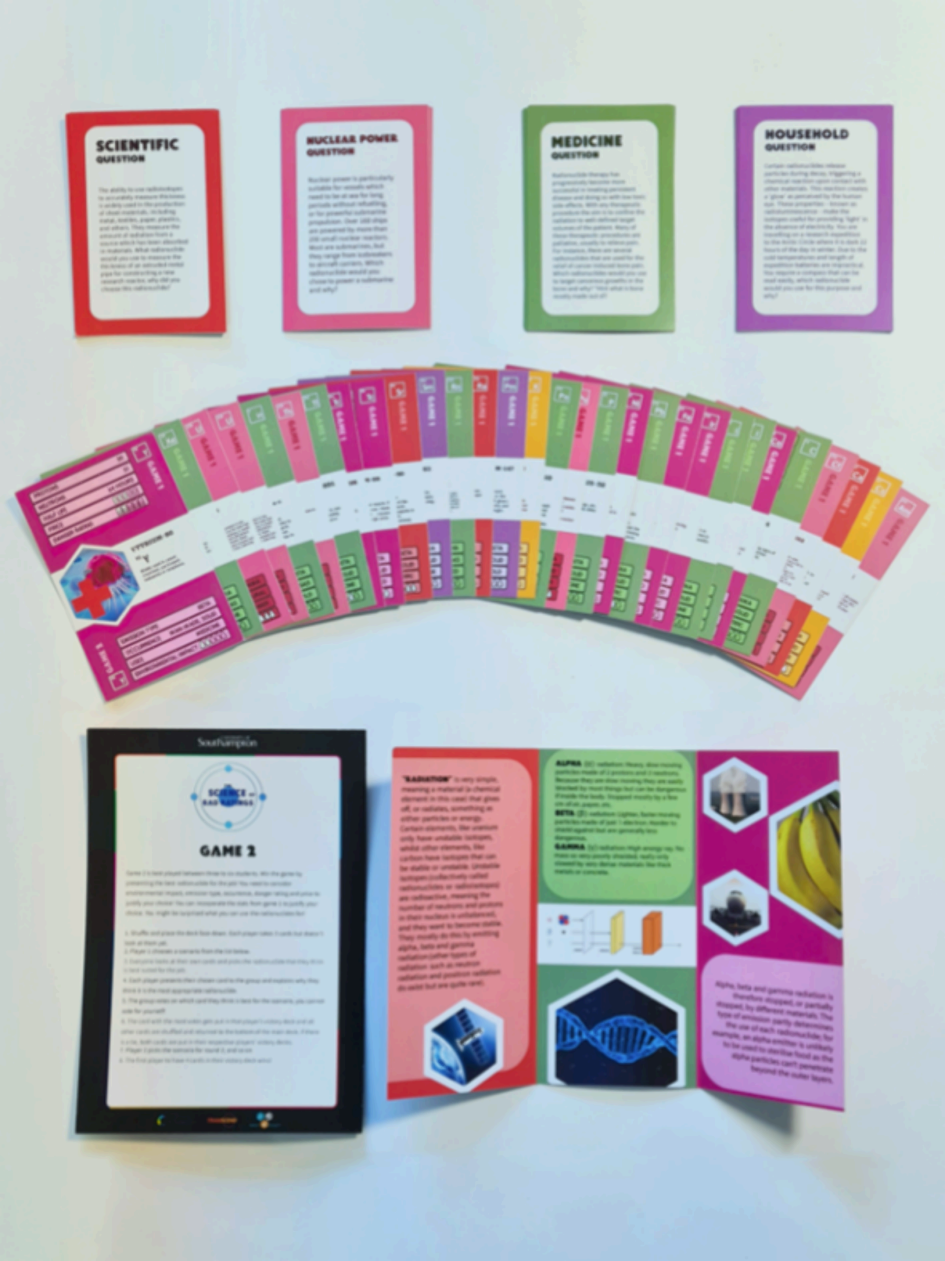
Example classroom set of *RAD Ratings*.

**Figure 2:**
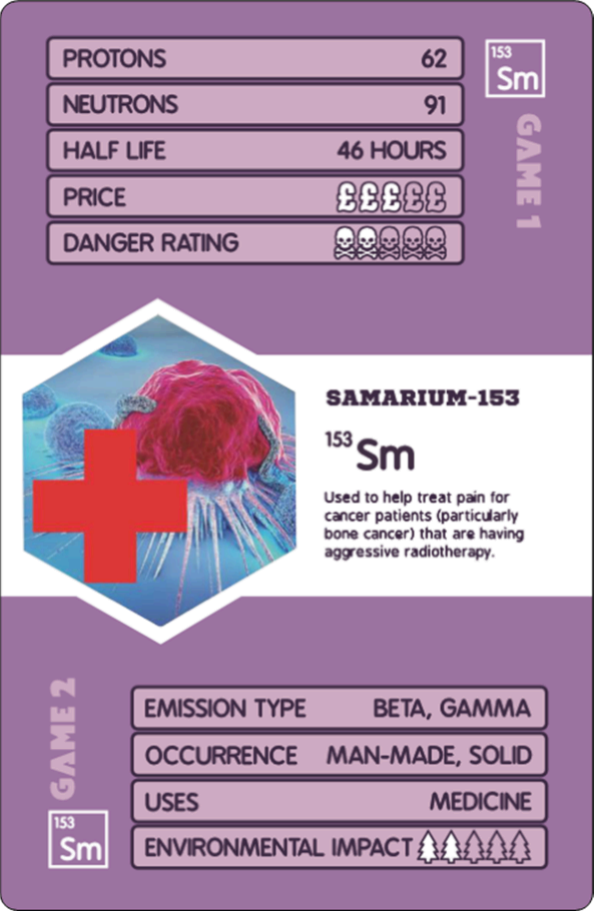
Example radionuclide card (samarium-153).

*RAD Ratings* (Figure 1) is a multiplayer interactive card game collaboratively developed by researchers at the University of Southampton, the Royal Society of Chemistry, and the TRANSCEND Consortium. Tailored as a supplementary teaching tool for students aged 13 and above in the UK, the game comprises 30 brightly colored, distinct radionuclide cards that illustrate wide-ranging applications within the nuclear industry. Activities and cards were designed to be seamlessly accommodated into a 50–60 min lesson timetable standard in English schools for the teaching of science subjects. Each card covers a radionuclide’s proton and neutron number, half-life, cost (relative to other isotopes), “danger” rating, decay type, occurrence, uses, and potential environmental impact (Figure 2) Each *RAD Ratings* pack can be used to play two games, both designed to accommodate a range of ages and learning abilities. Each card also includes example uses for each radionuclide.

Prior to the game play, students were introduced to the rules and mechanics of *RAD Ratings* through a standardized PowerPoint presentation provided to teachers. This introductory resource was included in the game pack that teachers received. Further details regarding the presentation and its content can be found in Supporting Information. This procedure ensures that all participants were familiar with the game’s objectives and rules before engaging in gameplay.

We have professionally designed, tested, and distributed over 400 card packs, complete with lesson plans and supporting classroom materials, throughout England to 13 schools and industry-linked STEM (Science, Technology, Engineering, and Mathematics) Ambassadors. Throughout 12 months of testing, these have reached more than 1,000 students. For STEM Ambassadors^a^ conducting in-person visits, preprepared card games have streamlined preparation and reduced barriers to previous school engagement.^21^

The objectives of *RAD Ratings* were to solidify the fundamental principles of the Key stage 4 (KS4) curriculum (ages 14 to 16), foster excitement, spark a broad interest in the field of nuclear science, and connect students with real-world uses of radioactivity, related career opportunities. KS4 in the UK National Curriculum is focused on preparing students for their General Certificate of Secondary Education (GCSE) examinations. It encompasses core subjects such as English, Mathematics, and Science, alongside optional subjects chosen based on students’ interests and future career paths.

### Game Description and Educational Objectives

Game 1 adopts a Top Trumps style format^b^, allowing students to compete using cost, half-life, proton/neutron numbers, and environmental impact relevant to the nuclear industry. This game can be completed in under 20 min, making it suitable for both introducing the fundamental concepts of radioactivity to younger students or as a preamble for more detailed teaching.

**Figure 3:**
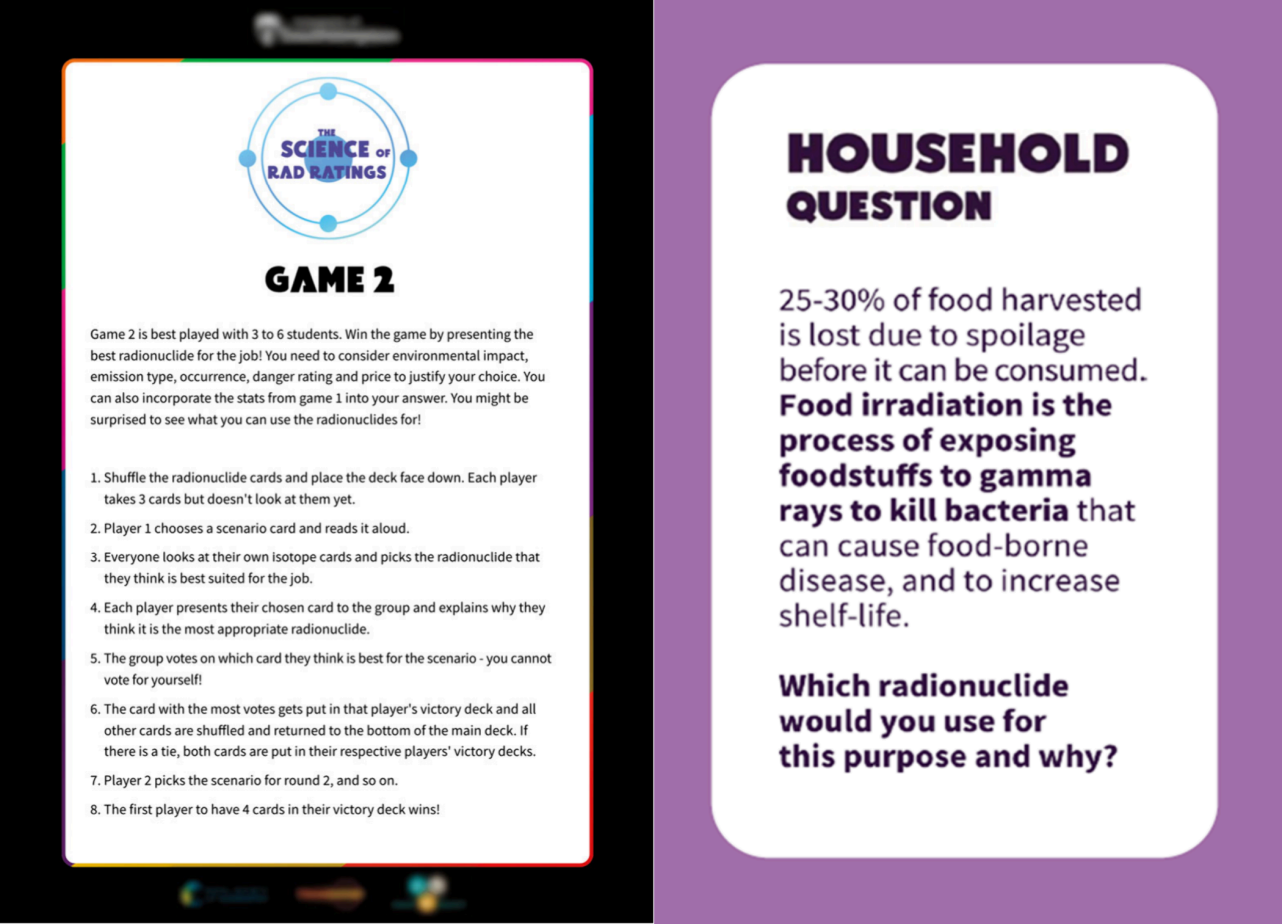
Game 1 instruction card on the left and example scenario card from game 2 on the right.

**Figure 4:**
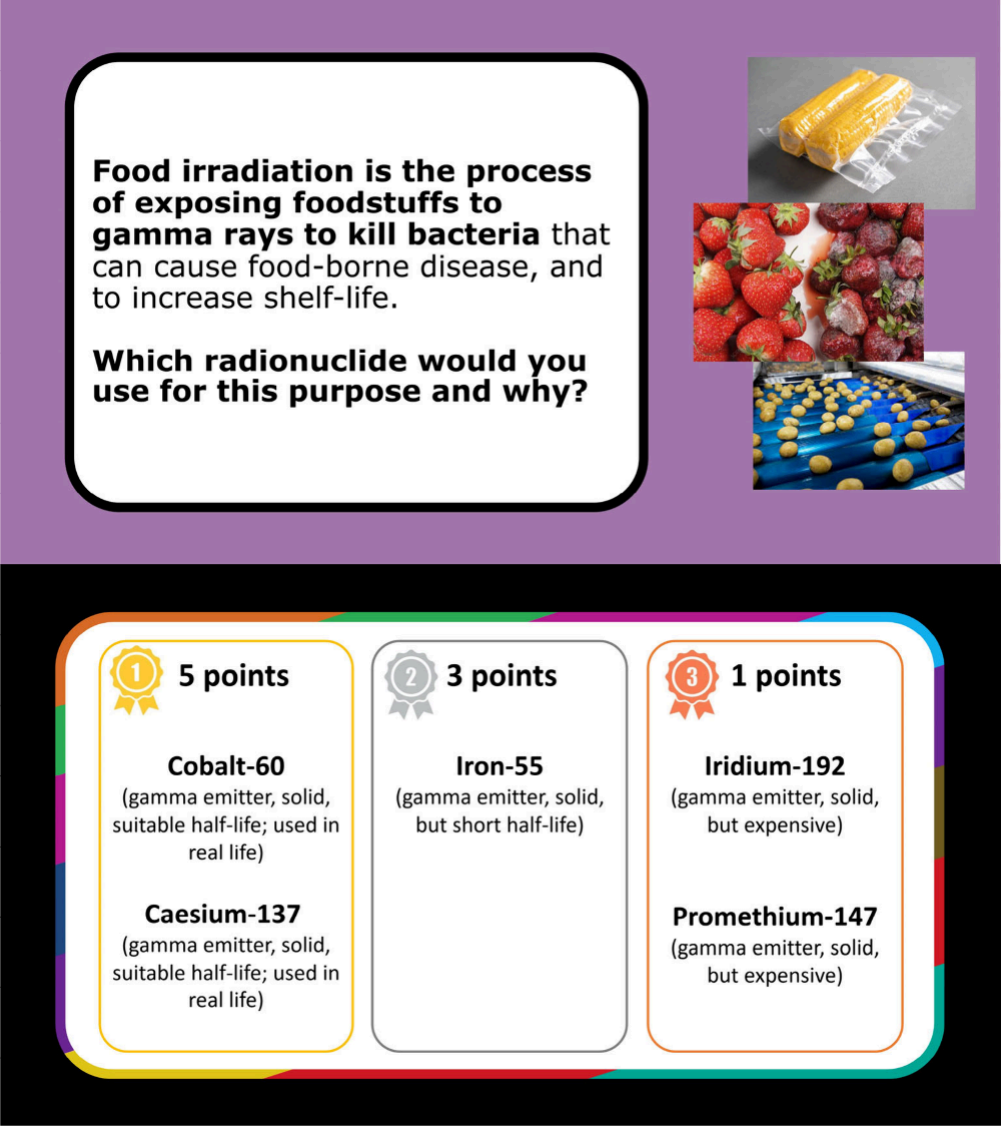
Example of Game 2 scenario PowerPoint question and answer.

Game 2 aims to highlight practical applications of nuclear science in daily life. Discussion points were crafted to encourage open-ended conversations, such as “*Which isotope would you use to power a satellite and why?*” (Figure 3). Although Game 2 is primarily discussion-based, the content has been adapted into a teacher-led quiz-style game, incorporating PowerPoint slides to facilitate whole-class participation and guided discussion (Figure 4). It is then up to teachers how they wish to make use of the game–either for a full lesson, or to supplement existing activities, revision, and so on. it is desirable for the discussions in game 2 to be led by someone with familiarity with radioactivity and the KS4 curriculum.

### Game Rules

Game 1 requires 2 to 5 players. Radionuclide cards are shuffled and dealt face down equally among all players. Players examine only their top card. Player 1 selects a category on the card and announces the chosen rating. Other players then call out the same category rating on their card; for example, in Figure 2, samarium-153 has a “Danger Rating” of 2. The player with the highest rating wins all of the cards used in that round, adding them to the bottom of their pile. In the case of a tie, cards are placed in a separate pile and the winner of the next round also adds these to their pile. The winner of each round selects the statistic for the next round, and the exercise repeats until one player collects all cards; this player is the winner.

Game 2 is best suited for 3 to 6 players. Each player collects 3 radionuclide cards from a central pile, face-down. Player 1 then chooses a scenario card and reads it aloud. Players then turn over their respective cards and select what they believe to be the most suitable card for that scenario. This is done by considering the environmental impact, emission type, occurrence, danger rating, cost, and half-life. Each player then takes turns explaining why their radionuclide is the best for the scenario. The group then votes on the most suitable card, and the winning card goes into that player’s victory deck. The cycle is repeated as desired; the player with the most cards in their deck at the end of the game is the winner.

## Teacher and Student Feedback

*RAD Ratings* was implemented in schools across rural and urban areas in England, followed by anonymous feedback collected via Microsoft Forms from participating students and teachers to gauge its success. Pre- and postactivity surveys for students assessed enjoyment and educational benefit related to radioactivity concepts. The surveys aimed to understand the impact of the game in stimulating student interest. Teacher surveys provided an overview of group engagement and benefits from the game, as well as corroborating student answers.

**Figure 5:**
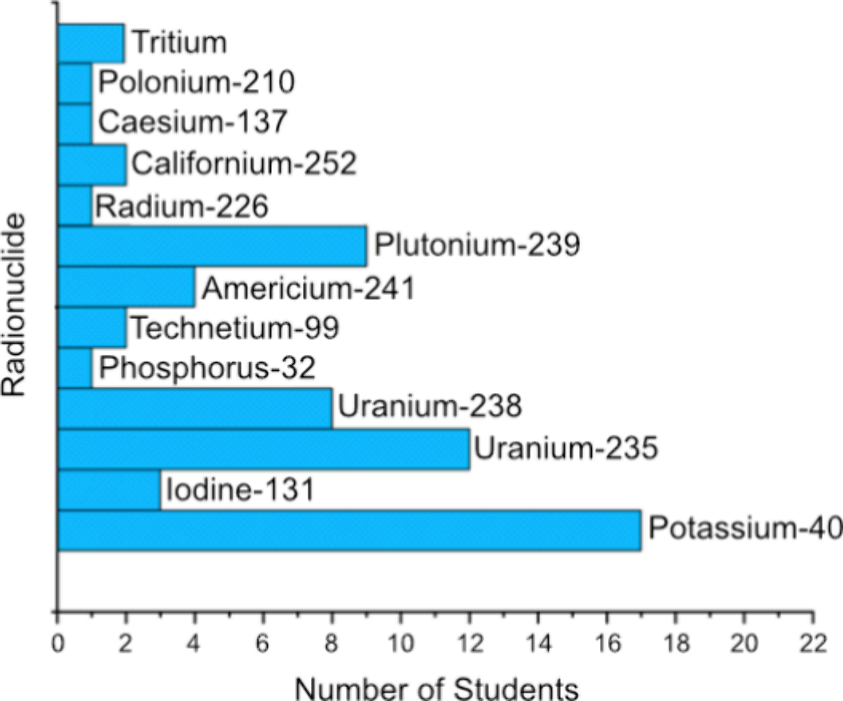
Student (*n* = 63) free form question on what their favorite radionuclide was after playing *RAD Ratings*.

A total of 98 students and 6 teachers were surveyed (Table 1 and Table 2). Questions included familiarity with the term “radionuclides”, understanding of the definition of radionuclides, examples of radionuclides and their uses, and an optional question about the students’ favorite radionuclides (Figure 5). This structured and free form approach aimed to measure *RAD Ratings*’ impact on student understanding and engagement with nuclear science concepts aligned with the Key Stage 4 curriculum.^22^

**Table 1:** Students Perceptions Before and After the Activity (*n* = 98)

Students Perceptions Before and After the Activity			
Question	Condition	Average	Standard Deviation
Had heard of the term ’radionuclides’.	Before	1.96	1.26
	After	2.86	1.32
Understood what radionuclides are.	Before	1.96	1.18
	After	2.66	1.25
Could give an example of a radionuclide and how it can be used.	Before	1.91	1.20
	After	2.66	1.25
Were interested in learning about radioactivity and its uses.	Before	3.01	1.21
	After	3.13	1.24

**Table 2:** Teachers Perceptions Before and After the Activity (*n* = 6)

Teachers Perceptions Before and After the Activity			
Question	Condition	Average	Standard Deviation
Had heard of the term ’radionuclides’.	Before	2.57	1.50
	After	3.43	1.18
Understood what radionuclides are.	Before	2.57	1.40
	After	3.14	0.99
Could give an example of a radionuclide and how it can be used.	Before	2.29	1.16
	After	3.00	1.20
Were interested in learning about radioactivity and its uses.	Before	3.00	1.51
	After	3.58	1.05

Before the activity, students demonstrated relatively low awareness of the term “radionuclides” (1.96, SD = 1.26) but improved to 2.86 (SD = 1.32) following the activity. Despite these improvements, the postactivity awareness scores for radionuclides still suggest that students are not fully familiar with the term. In terms of understanding, the students’ ability to grasp how radioactivity can be applied in different contexts improved from an average score of 3.20 (SD = 1.14) to 3.72 (SD = 1.25). This suggests that the activity contributed to enhancing their comprehension of the subject. Similarly, the understanding of radionuclides also showed improvement, rising from 1.96 (SD = 1.18) to 2.66 (SD = 1.25), though students’ understanding of this concept remained somewhat limited even after the activity. When it comes to practical application, such as providing examples of radionuclides, students’ abilities improved but still showed a considerable gap. Their average score increased from 1.91 (SD = 1.20) to 2.66 (SD = 1.25), which, while an improvement, still reflects challenges in applying this knowledge effectively. However, *n* = 63 students were able to provide an example of their favorite radionuclide. Finally, while there was a slight increase in students’ interest in learning about radioactivity and its uses (from 3.01 (SD = 1.21) to 3.13 (SD = 1.24)), the change was not as pronounced, suggesting that although the activity maintained their interest, it did not drastically increase it.

For teachers (Table 2), they assess the student’s awareness of the term “radionuclides” improved rising from 2.57 (SD = 1.50) to 3.43 (SD = 1.18), teachers also assessed student awareness of the term radionuclides to be higher 2.57 (SD = 1.50) compared to the student self-assessed awareness before the activity (1.96, SD = 1.26). In terms of comprehension, teachers’ assessed student understanding of how radioactivity can be applied in different contexts improved notably, with an increase in average score from 2.86 (SD = 1.25) to 3.57 (SD = 0.90). A similar trend was seen in the perceived student understanding of radionuclides, with the average score rising from 2.57 (SD = 1.40) to 3.14 (SD = 0.99).

Teachers also saw improvements in their student’s ability to provide examples of radionuclides, with the average score rising from 2.29 (SD = 1.16) to 3.00 (SD = 1.20), reflecting a positive shift in their practical application of the knowledge. Additionally, teachers reported an increase in student interest in the topic, with their average score rising from 3.00 (SD = 1.51) to 3.58 (SD = 1.05).

### Classroom Observations and Postactivity Interviews

Interviews with teachers and groups of students were conducted to further understand the strengths and limitations of the game. These were carried out either in person or online within 1 week of playing *RAD Ratings.* The results from these as well as from observations made throughout the sessions are summarized below:

#### Increased Interest in Radioactivity

Although student feedback showed only a slight increase in enthusiasm for learning about radioactivity and its practical applications after participating in RAD Ratings, these results were based on just one session of gameplay. In contrast, teachers reported a more significant shift in the interest in their responses to the survey. It is also worth considering that students may feel inclined to express favorable opinions about the game in person, while online surveys might elicit more candid feedback. This positive shift in interest was further supported by teachers’ observations.

#### Easy Implementation of Game 1

Feedback suggested that the simplicity of Game 1 made it a valuable addition to the classroom setting. Teachers noted that it supported and integrated with both KS3 (ages 11 to 14) and KS4 curricula, supporting existing lesson plans and making it a useful tool to reinforce key concepts related to radioactivity.

#### Game 1’s Suitability for Novices

Game 1 proved to be well-suited for students with limited prior knowledge of radioactivity. However, it was noted that this format had limitations in terms of expanding critical curriculum concepts, as it primarily focused on surface-level comparisons.

#### The Incorporation of a PowerPoint Presentation for Game 2

following teacher feedback that “Game 2 should be teacher-led”, a PowerPoint presentation was developed to accompany Game 2, transforming it into a whole-class activity. This format allowed for the rapid identification and addressing of areas where students lacked understanding or knowledge. Furthermore, teachers felt that they were better able to embed cross-curricular learning and revision, noting that direct parallels with additional science subjects were achievable. Despite the increased complexity, classroom observations and teacher feedback highlighted that Game 2 was more engaging than Game 1. Feedback suggests that the emphasis on applications and discussions of radioactivity concepts is a valuable tool for reinforcing students’ understanding across various science curriculum subjects.

#### Competitive Aspects of PowerPoint Presentation

Teacher feedback and classroom observations suggest that including the scoring system in the PowerPoint presentation for Game 2 introduced a competitive element, increasing student interest in the activity. Teachers observed that typically difficult-to-engage students participated well in the PowerPoint version of Game 2.

### Teacher and Student Experience

By assessing both teacher and student experiences, we gain a comprehensive view of how well the game aligns with educational objectives and gain a holistic understanding of the game’s impact. Both student and teacher experiences of *RAD Ratings* were assessed with online surveys using online Microsoft forms (one school provided feedback by printing off the online forms and filling them in) experience questions were included, the age ranges of the students filling in the forms were 14–17.

To assess the reception of *RAD Ratings* from students the postactivity survey included questions (Table 3) asking whether they whether they enjoyed playing *RAD Ratings* (average score of 3.79 (SD = 1.14) for enjoyment). The design of the game was also well-received, with students rating it 3.86 (SD = 1.03) and teachers giving it 3.83 (SD = 1.07) (Table 4). Both groups understood the instructions clearly, with average scores of 3.78 (SD = 1.11) for students and 3.83 (SD = 0.90) for teachers.

**Table 3:** What Students Think about RAD Ratings (*n* = 98)

What I Think About RAD Ratings (Students)		
Question	Average	Standard Deviation
I enjoyed playing Rad Ratings.	3.79	1.14
I understood the instructions for playing the game.	3.78	1.11
I like the design of the cards.	3.86	1.03
Playing the game helped me to better understand isotopes.	3.39	1.19
I would play this game again.	3.65	1.24

**Table 4:** What Teachers Think about Rad Ratings (*n* = 6)

What I Think About RAD Ratings (Teachers)		
Question	Average	Standard Deviation
The design of the cards is age appropriate.	3.83	1.07
The activity helped improve students’ critical thinking skills.	3.17	1.46
The activity helped improve students’ interest in the topic.	4.33	0.47
The activity helped students understand isotopes and their uses.	3.67	1.37
The activity was easy to implement in the classroom.	4.17	0.69

The activity was seen as valuable for educational purposes. Teachers felt that it helped improve students’ critical thinking (3.17, SD = 1.46) and increased students’ interest in the topic (4.33, SD = 0.47). Teachers also found the activity easy to implement in the classroom (4.17, SD = 0.69) and believed it improved students’ understanding of isotopes and their applications (3.67, SD = 1.37).

Explicit questions asking students to rate their enjoyment of the game was not asked in initial assessments of Radioactivity and U-235, and in the distribution of agree and strongly agree was similar to student feedback in Isotope Rummy.^12,20^

Our results reflected a similar positive distribution to the teacher feedback in Isotope Rummy, although in Isotope Rummy the teachers played the game themselves as if they were students rather than observing students playing the game in a classroom setting.^20^ Additionally, only undergraduate and graduate students were used to test Radioactivity and U-235 no teacher survey responses were recorded in that study.^12^ To address comments in postactivity interviews which included clearer visual instructions for both Game 1 and Game 2, the PowerPoint presentation with sequential instructions and information diagrams was created and distributed to all recipients of *RAD Ratings*.

## Conclusions

We have developed the card game *RAD Ratings*, distributed to 13 schools across England and reached over 1000 students in the first year, and tested the use of gamification to address the nuclear skills teaching gap in the UK. Additionally, the provision of 160 packs to industry-linked STEM Ambassadors in both public and private sectors underlines the potential for impact beyond the classroom. Student feedback reveals enhanced interest in radioactivity and its applications with >20% positive shift in student perception of radioactivity and >20% improvement in understanding of radionuclides and their uses following participation in *RAD Ratings*, corroborated by positive teacher feedback and classroom observations. Teacher comments emphasize the ease of implementation and the activity’s effectiveness as a learning resource.

A key observation has been the enhanced effectiveness of Game 2 with whole-class participation with teacher- and instructor-guided discussions compared to small groups playing Game 2 in isolation. Feedback on the whole-class participation approach, supported by the PowerPoint presentation, suggested that it enabled a structured framework for testing and improving their understanding of radioactivity and radionuclides and how these concepts are applied in everyday life. Furthermore, preprepared classroom packs with PowerPoint presentation the streamlined preparation process for STEM ambassadors highlights the practical benefits of gamified educational tools in outreach initiatives. This not only saves time but also lowers participation barriers, ensuring a smoother and more engaging experience for both ambassadors and students. Work is underway with both public and private sectors to develop this resource further, and we will report these results in due course.

## Supporting Information Available

Information leaflet, Game 1 instruction card, Game 2 instruction card, radionuclide cards, and scenario cards (PDF)
PowerPoint presentation for game (PDF)
Creation of the cards and game distribution (PDF)

## Footnotes

a A STEM Ambassador is a volunteer in the UK’s STEM Ambassador Program, coordinated by STEM Learning, which promotes education in science, technology, engineering, and mathematics. Ambassadors from diverse STEM backgrounds inspire young people through school visits, events, and hands-on activities, encouraging careers in STEM fields. (Source: STEM Learning, https://www.stem.org.uk/stem-ambassadors, accessed 2024.)

b Top Trumps is a card game where players compare numerical values across categories like speed or power. Players take turns selecting a category; the highest value wins the round. The game continues until one player has all the cards or a set number of rounds is completed.
