# Supplementary material for: Decked Out for Success: A Novel Card Game to Support School Teaching of Radioactivity and Nuclear Science
Source: J Chem Educ. 2024 Dec 18;102(1):430–6. doi: 10.1021/acs.jchemed.4c00603 (PMC11736787; doi:10.1021/acs.jchemed.4c00603)
Supplement: Supplementary file 1 — ed4c00603_si_001.pdf [file ed4c00603_si_001.pdf]

Supplementary information 1

Decked out for success: a novel card game to support school teaching of radioactivity and nuclear science

Sarah E. Lu[1*], Shaun D. Hemming[1], Jamie M. Purkis[1]
[1]University of Southampton, University Road, Southampton SO17 1BJ, United Kingdom (UK)
*Corresponding author: Sarah Lu, Sarah.Lu@soton.ac.uk

Contents:

Information leaflet
Game 1 instruction card
Game 2 instruction card
Radionuclide cards
Scenario cards

THE SCIENCE OF RAD RATINGS

GAME 1

Game 1 can be played with a minimum of 2 students and a maximum of 5 students.

1. Shuffle and deal the radionuclide cards face down between all players.
2. Players pick up their cards and look at their top cards only.
3. Player 1 chooses a question [examples are written below] and calls out their best rating.
4. The other players see if they can beat this rating.
5. The card with the best rating wins all of the top cards and adds them to the base of their pile. If players tie (e.g. they have the same danger rating) then all top cards are put in a new pile and the winner of the next round gets to keep them.
6. The winner of the round chooses the stat for the next round.
7. The first player to collect all of the cards wins!

Example questions: Which radionuclide...

- has the longest half life?
- has the highest number of protons?
- has the highest number of neutrons?
- has the shortest half-life?
- is the cheapest?
- is the most expensive?
- is the least dangerous?
- is the most dangerous?

THE SCIENCE OF RAD RATINGS

GAME 2

Game 2 is best played with 3 to 6 students. Win the game by presenting the best radionuclide for the job! You need to consider environmental impact, emission type, occurrence, danger rating and price to justify your choice. You can also incorporate the stats from game 1 into your answer. You might be surprised to see what you can use the radionuclides for!

1. Shuffle the radionuclide cards and place the deck face down. Each player takes 3 cards but doesn't look at them yet.
2. Player 1 chooses a scenario card and reads it aloud.
3. Everyone looks at their own radionuclide cards and picks the isotope that they think is best suited for the job.
4. Each player presents their chosen card to the group and explains why they think it is the most appropriate radionuclide.
5. The group votes on which card they think is best for the scenario - you cannot vote for yourself!
6. The card with the most votes gets put in that player's victory deck and all other cards are shuffled and returned to the bottom of the main deck. If there is a tie, both cards are put in their respective players' victory decks.
7. Player 2 picks the scenario for round 2, and so on.
8. The first player to have 4 cards in their victory deck wins!

AMERICIUM-241

GAME 1 — 241 Am

PROTONS	95
NEUTRONS	146
HALF LIFE	433 YEARS
PRICE	££££££
DANGER RATING	

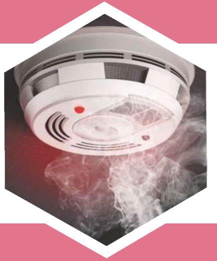

^{241}Am

Used in fire prevention; nearly all smoke detectors sold in the UK in the last 50+ years contain very small amounts of americium-241, including ones in your home!

GAME 2 — 241 Am

EMISSION TYPE	ALPHA, GAMMA
OCCURRENCE	MAN-MADE, SOLID
USES	HOUSEHOLD
ENVIRONMENTAL IMPACT	

CAESIUM-137

GAME 1 — 137 Cs

PROTONS	55
NEUTRONS	82
HALF LIFE	30 YEARS
PRICE	££££££
DANGER RATING	

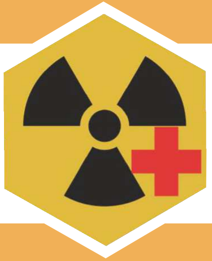

^{137}Cs

Caesium-137 is used in medical radiation therapy devices for treating cancer, as well as for measuring the thickness of various materials.

GAME 2 — 137 Cs

EMISSION TYPE	BETA, GAMMA
OCCURRENCE	MAN-MADE, SOLID
USES	MEDICINE
ENVIRONMENTAL IMPACT	

CALCIUM-47

GAME 1 — 47 Ca

PROTONS	20
NEUTRONS	27
HALF LIFE	4.5 DAYS
PRICE	££££££
DANGER RATING	

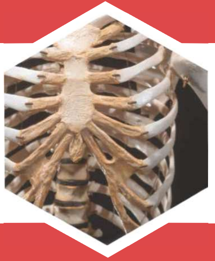

^{47}Ca

Calcium-47 is used in medicine and research to investigate bone metabolism problems or to diagnose calcium disorders (bones are made of calcium phosphate).

GAME 2 — 47 Ca

EMISSION TYPE	BETA, GAMMA
OCCURRENCE	MAN-MADE, SOLID
USES	MEDICINE
ENVIRONMENTAL IMPACT	

CALIFORNIUM-252

GAME 1 — 252 Cf

PROTONS	98
NEUTRONS	154
HALF LIFE	2.6 YEARS
PRICE	£££££
DANGER RATING	

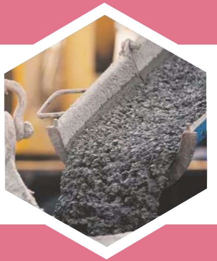

^{252}Cf

Californium-252 is used commercially for determining the water content in coals, cements and minerals. It is also an alternative to nickel-63 for detecting and identifying explosives, landmines, and unexploded military ordinance.

GAME 2 — 252 Cf

EMISSION TYPE	ALPHA
OCCURRENCE	MAN-MADE, SOLID
USES	SCIENTIFIC
ENVIRONMENTAL IMPACT	

CARBON-14

GAME 1 — 14 C

PROTONS	6
NEUTRONS	8
HALF LIFE	5700 YEARS
PRICE	£££££
DANGER RATING	

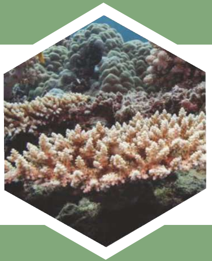

^{14}C

Useful for determining the ages of artefacts that are between 500 and 50,000 years old (radiocarbon dating uses carbon-14).

GAME 2 — 14 C

EMISSION TYPE	BETA, GAMMA
OCCURRENCE	NATURAL, SOLID
USES	SCIENTIFIC
ENVIRONMENTAL IMPACT	

COBALT-60

GAME 1 — 60 Co

PROTONS	27
NEUTRONS	33
HALF LIFE	5 YEARS
PRICE	£££££
DANGER RATING	

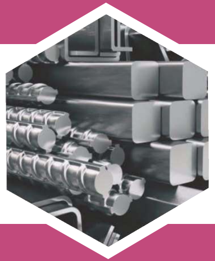

^{60}Co

Widely used for analysing metal welds, cancer treatment, and food sterilisation (the gamma emissions kill bacteria, making food last longer).

GAME 2 — 60 Co

EMISSION TYPE	BETA, GAMMA
OCCURRENCE	MAN-MADE, SOLID
USES	MEDICINE
ENVIRONMENTAL IMPACT	

IODINE-123

GAME 1 — 123 I

PROTONS	53
NEUTRONS	70
HALF LIFE	13 HOURS
PRICE	£££££
DANGER RATING	

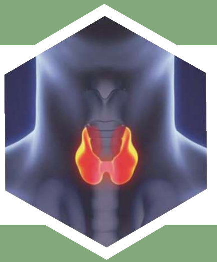

^{123}I

Used to image and diagnose problems with thyroid glands. The thyroid helps control metabolism, growth and development.

GAME 2 — 123 I

EMISSION TYPE	BETA
OCCURRENCE	MAN-MADE, SOLID
USES	MEDICINE
ENVIRONMENTAL IMPACT	

IODINE-131

GAME 1 — 131 I

PROTONS	53
NEUTRONS	78
HALF LIFE	8 DAYS
PRICE	£££££
DANGER RATING	

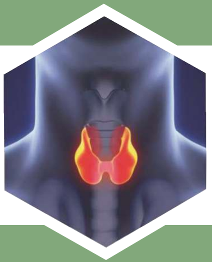

^{131}I

Used in medicine to treat cancerous and non-cancerous (benign) conditions of the thyroid gland.

GAME 2 — 131 I

EMISSION TYPE	BETA, GAMMA
OCCURRENCE	MAN-MADE, SOLID
USES	MEDICINE
ENVIRONMENTAL IMPACT	

IRIDIUM-192

GAME 1 — 192 Ir

PROTONS	77
NEUTRONS	115
HALF LIFE	74 DAYS
PRICE	£££££
DANGER RATING	

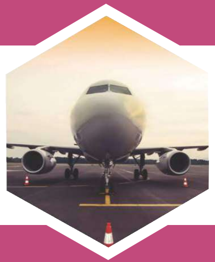

^{192}Ir

Used in aeroplane engineering to test how well a plane is welded together.

GAME 2 — 192 Ir

EMISSION TYPE	BETA, GAMMA
OCCURRENCE	MAN-MADE, SOLID
USES	SCIENTIFIC
ENVIRONMENTAL IMPACT	

GAME 1 — ^{55}Fe

PROTONS	26
NEUTRONS	29
HALF LIFE	3 YEARS
PRICE	
DANGER RATING	

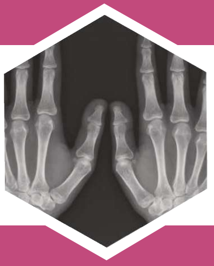

IRON-55

^{55}Fe

Used to generate X-rays for analysing scientific samples. Iron-55 atoms emit X-rays when they decay, so no electrical power is needed for the devices to work.

GAME 2 — ^{55}Fe

EMISSION TYPE	BETA
OCCURRENCE	MAN-MADE, SOLID
USES	SCIENTIFIC
ENVIRONMENTAL IMPACT	

GAME 1 — ^{210}Pb

PROTONS	82
NEUTRONS	128
HALF LIFE	22 YEARS
PRICE	
DANGER RATING	

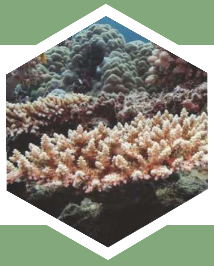

LEAD-210

^{210}Pb

Used to determine how old soils, rocks and other environmental things are, particularly from lakes and the sea floor. Lead-210 dating works on items that are between 2 and 150 years old.

GAME 2 — ^{210}Pb

EMISSION TYPE	BETA, GAMMA
OCCURRENCE	NATURAL, SOLID
USES	SCIENTIFIC
ENVIRONMENTAL IMPACT	

GAME 1 — ^{63}Ni

PROTONS	28
NEUTRONS	35
HALF LIFE	100 YEARS
PRICE	
DANGER RATING	

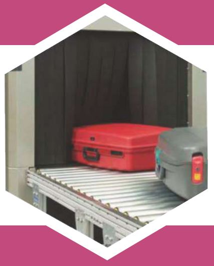

NICKEL-63

^{63}Ni

Used both in airport scanners to detect potential explosives, and in scientific analysis.

GAME 2 — ^{63}Ni

EMISSION TYPE	BETA
OCCURRENCE	MAN-MADE, SOLID
USES	SCIENTIFIC
ENVIRONMENTAL IMPACT	

GAME 1 — ^{32}P

PROTONS	15
NEUTRONS	17
HALF LIFE	14 DAYS
PRICE	
DANGER RATING	

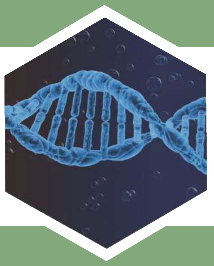

PHOSPHORUS-32

^{32}P

Used in genetic research to track DNA (DNA contains phosphorus). Also used in plant science to monitor fertiliser absorption.

GAME 2 — ^{32}P

EMISSION TYPE	BETA
OCCURRENCE	MAN-MADE, SOLID
USES	MEDICINE
ENVIRONMENTAL IMPACT	

GAME 1 — ^{238}Pu

PROTONS	94
NEUTRONS	144
HALF LIFE	88 YEARS
PRICE	
DANGER RATING	

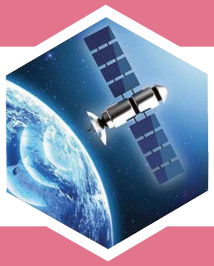

PLUTONIUM-238

^{238}Pu

Probably the most dangerous element in the periodic table. Extremely radioactive and toxic, but also very useful; it can power spaceship engines (radioisotope thermoelectric generators), and can be used in nuclear weapons and power.

GAME 2 — ^{238}Pu

EMISSION TYPE	ALPHA
OCCURRENCE	MAN-MADE, SOLID
USES	NUCLEAR POWER
ENVIRONMENTAL IMPACT	

GAME 1 — ^{210}Po

PROTONS	84
NEUTRONS	126
HALF LIFE	138 DAYS
PRICE	
DANGER RATING	

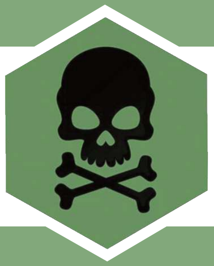

POLONIUM-210

^{210}Po

Used in anti-static brushes to remove dust from photographic film (in much higher doses it was used to assassinate Alexander Litvinenko in 2006!).

GAME 2 — ^{210}Po

EMISSION TYPE	ALPHA
OCCURRENCE	MAN-MADE, SOLID
USES	SCIENTIFIC
ENVIRONMENTAL IMPACT	

GAME 1 — ^{40}K

PROTONS	19
NEUTRONS	21
HALF LIFE	1.3 BILLION YEARS
PRICE	
DANGER RATING	

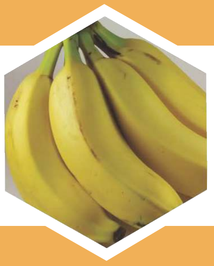

POTASSIUM-40

^{40}K

Most of the radioactivity in humans comes from potassium-40. It is also present in bananas (but you'd need to eat about 50 million bananas in one go to die from the radiation!).

GAME 2 — ^{40}K

EMISSION TYPE	BETA, GAMMA
OCCURRENCE	NATURAL, SOLID
USES	HOUSEHOLD
ENVIRONMENTAL IMPACT	

GAME 1 — ^{147}Pm

PROTONS	61
NEUTRONS	86
HALF LIFE	2.6 YEARS
PRICE	
DANGER RATING	

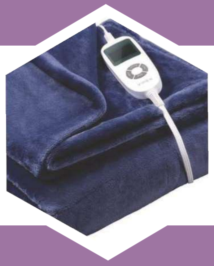

PROMETHIUM-147

^{147}Pm

Historically used to ensure electric blankets stay at the correct temperature. It glows thanks to its radioactivity and so it is sometimes used in night-vision sniper scopes.

GAME 2 — ^{147}Pm

EMISSION TYPE	BETA
OCCURRENCE	MAN-MADE, SOLID
USES	SCIENTIFIC
ENVIRONMENTAL IMPACT	

GAME 1 — ^{226}Ra

PROTONS	88
NEUTRONS	138
HALF LIFE	1600 YEARS
PRICE	
DANGER RATING	

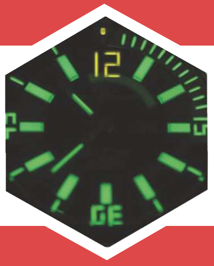

RADIUM-226

^{226}Ra

Used in the past to make glow-in-the-dark watches. Marie and Pierre Curie first helped to study the radioactivity of radium in the late 1800s and into the 1900s.

GAME 2 — ^{226}Ra

EMISSION TYPE	ALPHA, GAMMA
OCCURRENCE	NATURAL, SOLID
USES	HOUSEHOLD (IN THE PAST)
ENVIRONMENTAL IMPACT	

RADON-222

GAME 1	^{222}Rn
PROTONS	86
NEUTRONS	136
HALF LIFE	4 DAYS
PRICE	£££££
DANGER RATING	

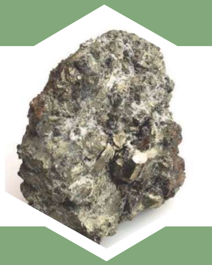

RADON-222

^{222}Rn

A naturally-occurring radioactive gas, radon can sometimes be a problem in areas with a lot of granite (which gives off radon). Some homes are fitted with radon sensors to detect if the levels of radon are getting too high.

GAME 2	^{222}Rn
EMISSION TYPE	ALPHA
OCCURRENCE	NATURAL, GAS
USES	NOT USED
ENVIRONMENTAL IMPACT	

SAMARIUM-153

GAME 1	^{153}Sm
PROTONS	62
NEUTRONS	91
HALF LIFE	46 HOURS
PRICE	£££££
DANGER RATING	

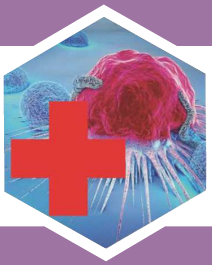

SAMARIUM-153

^{153}Sm

Used to help treat pain for cancer patients (particularly bone cancer) that are having aggressive radiotherapy.

GAME 2	^{153}Sm
EMISSION TYPE	BETA, GAMMA
OCCURRENCE	MAN-MADE, SOLID
USES	MEDICINE
ENVIRONMENTAL IMPACT	

STRONTIUM-90

GAME 1	^{90}Sr
PROTONS	38
NEUTRONS	52
HALF LIFE	28 YEARS
PRICE	£££££
DANGER RATING	

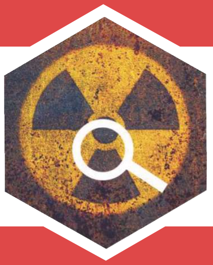

STRONTIUM-90

^{90}Sr

Used to detect nuclear weapons testing (one of the ways we know how North Korea tests nuclear weapons is to look for atmospheric strontium-90 concentrations).

GAME 2	^{90}Sr
EMISSION TYPE	BETA
OCCURRENCE	MAN-MADE, SOLID
USES	SCIENTIFIC
ENVIRONMENTAL IMPACT	

TECHNETIUM-99

GAME 1	^{99}Tc
PROTONS	43
NEUTRONS	56
HALF LIFE	211,000 YEARS
PRICE	£££££
DANGER RATING	

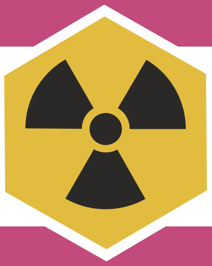

TECHNETIUM-99

^{99}Tc

A big problem in nuclear waste. If it gets into water it can move great distances and causes contamination over a large area.

GAME 2	^{99}Tc
EMISSION TYPE	BETA
OCCURRENCE	MAN-MADE, SOLID
USES	NOT USED
ENVIRONMENTAL IMPACT	

TECHNETIUM-99M

GAME 1	^{99m}Tc
PROTONS	43
NEUTRONS	56
HALF LIFE	6 HOURS
PRICE	£££££
DANGER RATING	

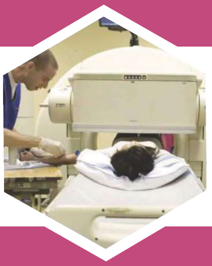

TECHNETIUM-99M

^{99m}Tc

The most widely used radioisotope in radiation-based medical treatments and imaging. Used in procedures involving the brain, bones, liver, kidneys etc.

GAME 2	^{99m}Tc
EMISSION TYPE	GAMMA
OCCURRENCE	MAN-MADE, SOLID
USES	MEDICINE
ENVIRONMENTAL IMPACT	

THALLIUM-201

GAME 1	^{201}Tl
PROTONS	81
NEUTRONS	120
HALF LIFE	3 DAYS
PRICE	£££££
DANGER RATING	

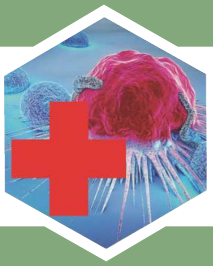

THALLIUM-201

^{201}Tl

Used as a radiotracer to see how much blood is reaching the different parts of your heart.

GAME 2	^{201}Tl
EMISSION TYPE	BETA, GAMMA
OCCURRENCE	MAN-MADE, SOLID
USES	MEDICINE
ENVIRONMENTAL IMPACT	

THORIUM-232

GAME 1	^{232}Th
PROTONS	90
NEUTRONS	142
HALF LIFE	14 BILLION YEARS
PRICE	£££££
DANGER RATING	

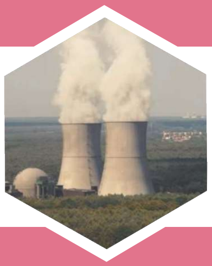

THORIUM-232

^{232}Th

Used as an alternative to uranium in some nuclear power stations.

GAME 2	^{232}Th
EMISSION TYPE	ALPHA
OCCURRENCE	NATURAL, SOLID
USES	NUCLEAR POWER
ENVIRONMENTAL IMPACT	

TRITIUM

GAME 1	^{3}H
PROTONS	1
NEUTRONS	2
HALF LIFE	12 YEARS
PRICE	£££££
DANGER RATING	

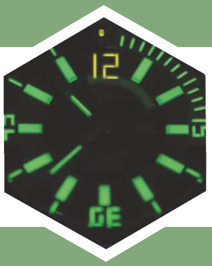

TRITIUM (HYDROGEN-3)

^{3}H

Used in everything from glow-in-the-dark watches to nuclear weapons research. Among the most common radioisotopes used in day-to-day life.

GAME 2	^{3}H
EMISSION TYPE	BETA
OCCURRENCE	NATURAL, LIQUID
USES	HOUSEHOLD
ENVIRONMENTAL IMPACT	

URANIUM-235

GAME 1	^{235}U
PROTONS	92
NEUTRONS	143
HALF LIFE	700 MILLION YEARS
PRICE	£££££
DANGER RATING	

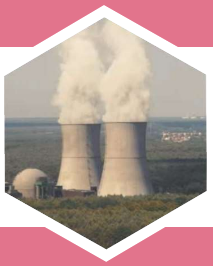

URANIUM-235

^{235}U

Although only about 1% of uranium in the world, uranium-235 is used as the main fuel source for nuclear power stations. It needs to be separated from uranium-238 before use, through uranium enrichment.

GAME 2	^{235}U
EMISSION TYPE	ALPHA, GAMMA
OCCURRENCE	NATURAL, SOLID
USES	NUCLEAR POWER
ENVIRONMENTAL IMPACT	

GAME 1	238 U
PROTONS	92
NEUTRONS	146
HALF LIFE	4.5 BILLION YEARS
PRICE	£££££
DANGER RATING	

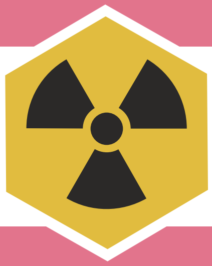

URANIUM-238

^{238}U

About 99% of uranium in the world is uranium-238. It cannot be used as nuclear reactor fuel and so remains largely purposeless, although it is occasionally made into armour-piercing weapons for the military.

GAME 2	238 U
EMISSION TYPE	ALPHA, GAMMA
OCCURRENCE	NATURAL, SOLID
USES	NUCLEAR POWER
ENVIRONMENTAL IMPACT	

GAME 1	133 Xe
PROTONS	54
NEUTRONS	79
HALF LIFE	5 DAYS
PRICE	£££££
DANGER RATING	

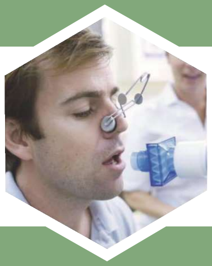

XENON-133

^{133}Xe

Xenon-133 is a gas that can be inhaled and used to image the lungs to see how well they are working.

GAME 2	133 Xe
EMISSION TYPE	BETA, GAMMA
OCCURRENCE	MAN-MADE, GAS
USES	MEDICINE
ENVIRONMENTAL IMPACT	

GAME 1	90 Y
PROTONS	39
NEUTRONS	51
HALF LIFE	64 HOURS
PRICE	£££££
DANGER RATING	

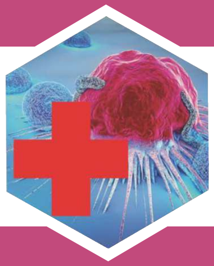

YTTRIUM-90

^{90}Y

Widely used in cancer treatment, particularly with tumours in the liver.

GAME 2	90 Y
EMISSION TYPE	BETA
OCCURRENCE	MAN-MADE, SOLID
USES	MEDICINE
ENVIRONMENTAL IMPACT	

HOUSEHOLD QUESTION

25-30% of food harvested is lost due to spoilage before it can be consumed. **Food irradiation is the process of exposing foodstuffs to gamma rays to kill bacteria** that can cause food-borne disease, and to increase shelf-life.

Which radionuclide would you use for this purpose and why?

HOUSEHOLD QUESTION

One of the most common uses of radioisotopes today is in household smoke detectors. **Smoke detectors work by a radionuclide emitting particles which ionise the air** and allows a current to flow between two electrodes.

Which is the best radionuclide to use for this purpose and why?

HOUSEHOLD QUESTION

Certain radionuclides release particles during decay, triggering a chemical reaction upon contact with other materials and creating a glow. This reaction is known as **radioluminescence.**

You are travelling on a research expedition to the Arctic Circle in winter where it is dark 22 hours of the day. **You require a compass that can be read easily, which radionuclide would you use for this purpose and why?**

MEDICINE QUESTION

Diagnostic techniques in nuclear medicine use radiopharmaceuticals (or radiotracers) which emit particles or rays from within the body.

These tracers are generally short-lived isotopes, and their emissions are detected by an imaging device.

A patient needs to have the gas exchange in their lungs investigated, which radionuclide would you use for these investigations and why?

MEDICINE QUESTION

Nuclear medicine is also used for therapeutic purposes.

Which radionuclide would you use to treat a cancerous tumour affecting the thyroid gland?

Please explain your choice!

MEDICINE QUESTION

With any therapeutic procedure, the aim is to confine the radiation to well-defined target volumes of the patient.

There are several radionuclides that are used for treating bone cancer.

Which radionuclides would you use to target cancerous growths in the bone and why?

NUCLEAR POWER QUESTION

Radioisotope Thermoelectric Generators (RTGs) have been used as an electricity source in spacecraft since 1961 **due to their high decay heat and short range of particle emissions.**

You are in charge of selecting an appropriate radionuclide to power a **50-year satellite mission to Mars, which radionuclide do you choose to power your satellite and why?**

NUCLEAR POWER QUESTION

Advanced Gas-cooled Reactors (AGRs) are the second generation of British gas-cooled reactors.

These reactors, like other nuclear technology, use the energy released by splitting atoms of certain elements.

Which is the best radionuclide to use as a fuel for an AGR and why?

NUCLEAR POWER QUESTION

Nuclear power is particularly suitable for vessels which need to be at sea for long periods without refuelling, or for powerful submarine propulsion.

Which radionuclide would you chose to power a submarine and why?

SCIENTIFIC
QUESTION

Efficient use of fertilisers is a concern to both developing and developed countries.

Fertilisers 'labelled' with a particular isotope provides a means of **finding out how much fertiliser is taken up by the plant** and how much is lost into the environment.

Which radionuclide would you use for this purpose and why?

SCIENTIFIC
QUESTION

Analysing naturally-occurring radioisotopes is important for **determining the age of rocks and other materials** that are of interest to geologists, anthropologists, and archaeologists, among others.

A historian thinks the wooden beams in an old house were made in the early Tudor period (about **500 years ago**). **Which radionuclide would you use to check the age of the wood**, and therefore the age of the house?

SCIENTIFIC
QUESTION

Radioisotopes can be used to **accurately measure the thickness of many different sheet materials.**

This is done by measuring the amount of radiation present before and after it passes through the material.

Which radionuclide would you use to **measure the thickness of an extruded metal pipe, why did you choose this radionuclide?**
