# Supplementary material for: Decked Out for Success: A Novel Card Game to Support School Teaching of Radioactivity and Nuclear Science
Source: J Chem Educ. 2024 Dec 18;102(1):430–6. doi: 10.1021/acs.jchemed.4c00603 (PMC11736787; doi:10.1021/acs.jchemed.4c00603)
Supplement: Supplementary file 2 — ed4c00603_si_002.pdf [file ed4c00603_si_002.pdf]

Supplementary information 2

Decked out for success: a novel card game to support school teaching of radioactivity and nuclear science

Sarah E. Lu[1*], Shaun D. Hemming[1], Jamie M. Purkis[1]
[1]University of Southampton, University Road, Southampton SO17 1BJ, United Kingdom (UK)
*Corresponding author: Sarah Lu, Sarah.Lu@soton.ac.uk

Contents:

PowerPoint presentation to accompany Game 2

THE SCIENCE OF RAD RATINGS

Classroom Presentation

University of Southampton

TRANSCEND

NNUF EXACT

ROYAL SOCIETY OF CHEMISTRY

The Winner Takes It All (Game 1)

How to play:

- In groups of 2-5, shuffle and deal the radionuclide cards face down equally between players.
- Players pick up their cards and look at their top cards only.

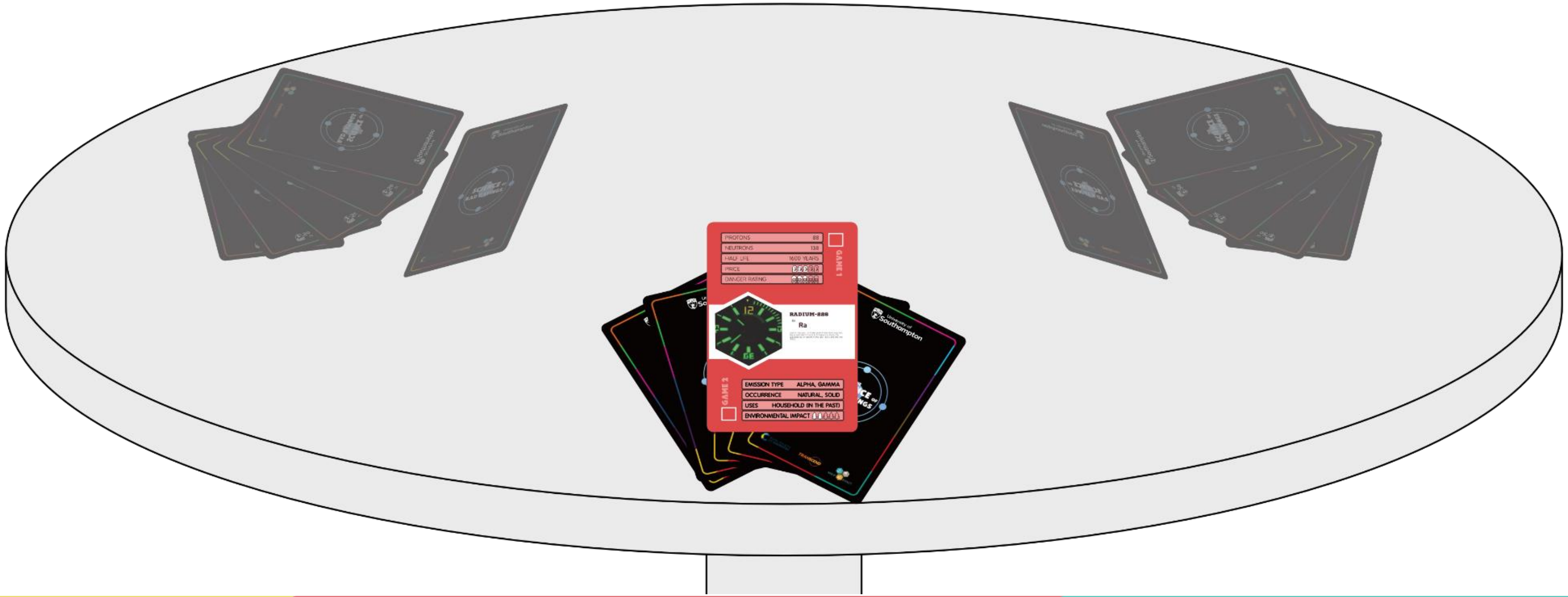

How to play:

- Player 1 chooses a question based on the stats at the top of each card (examples are on slide 7) and calls out their best rating.
- The other players see if they can beat this rating.

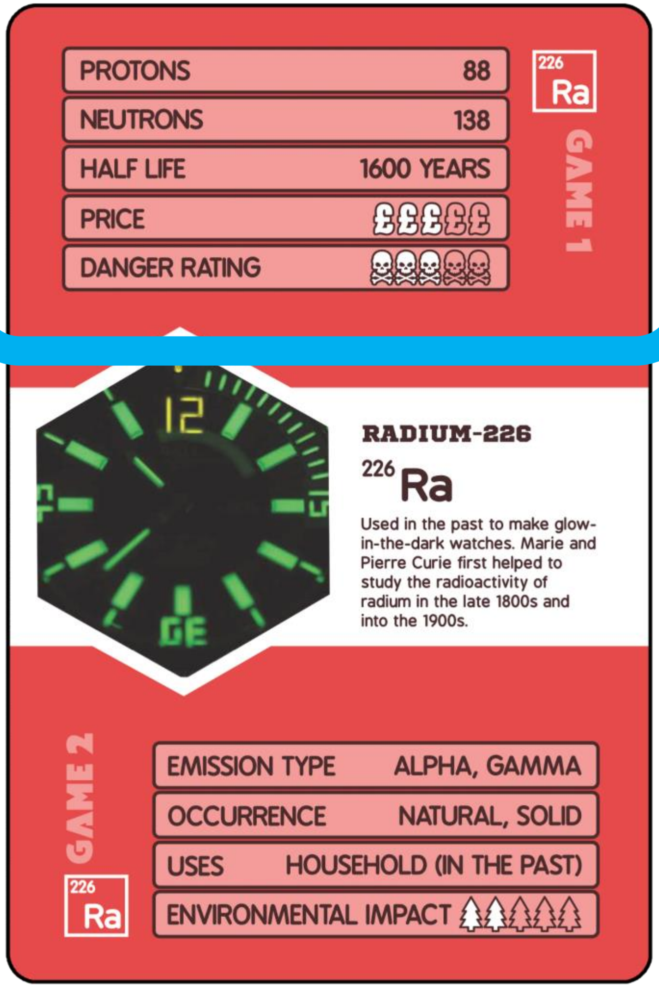

How to play:

- The card with the best rating wins all of the top cards and adds them to the base of their pile. If players tie (e.g. they have the same danger rating) then all top cards are put in a new pile and the winner of the next round gets to keep them.

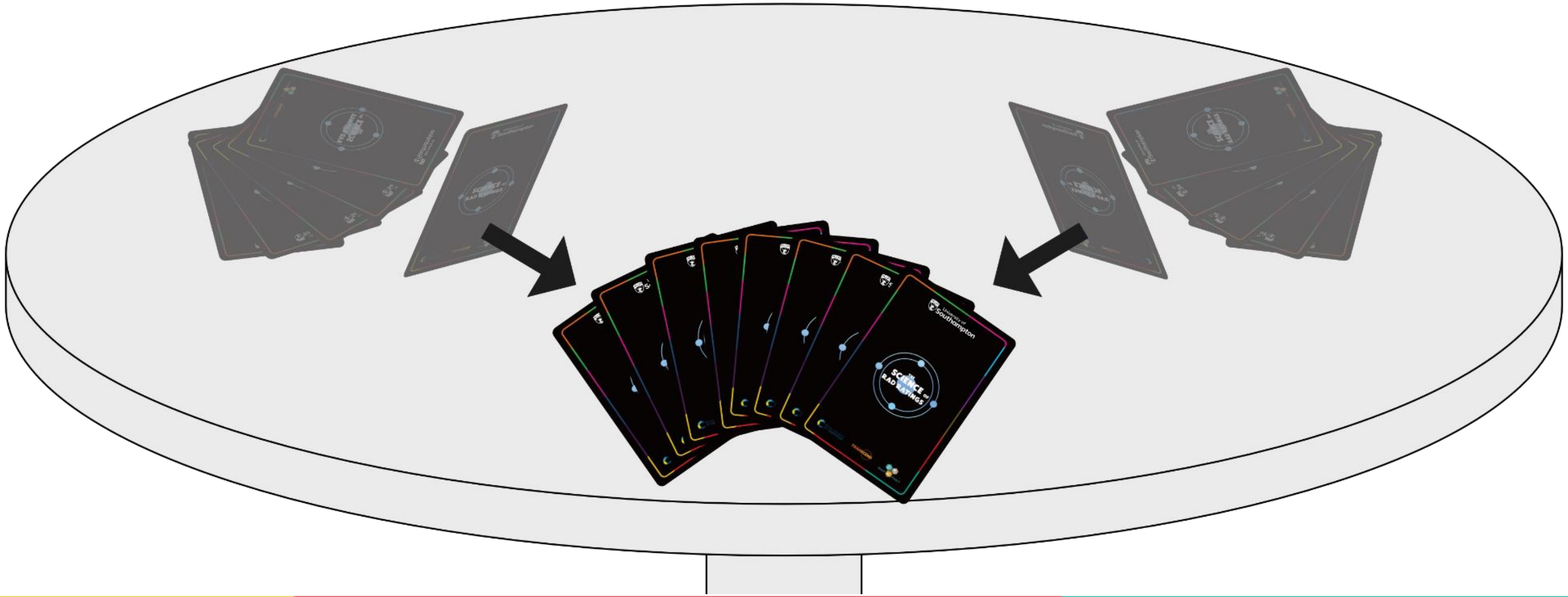

How to play:

- The winner of the round chooses the stat for the next round.
- The first player to collect all the cards wins!

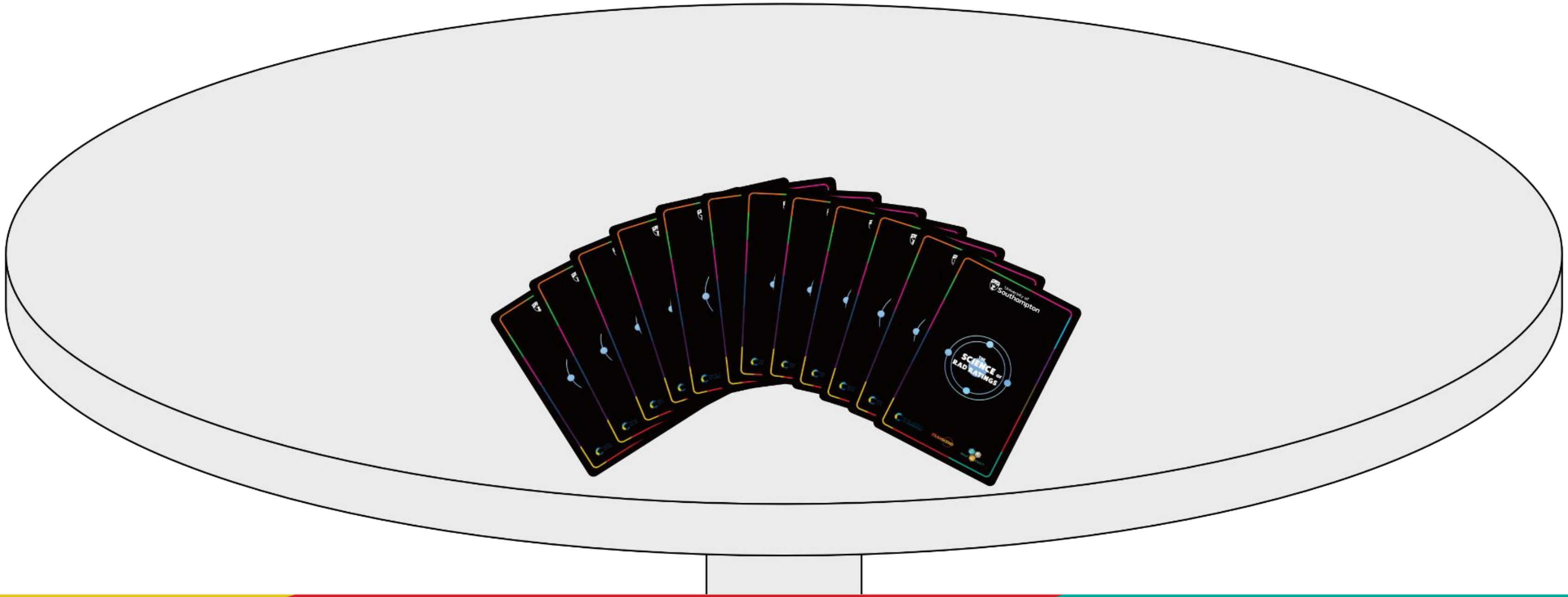

Example categories: Which radionuclide…

- Has the longest half-life?
- Has the highest number of protons?
- Is the least dangerous?
- Has the highest number of neutrons?
- Has the shortest half life?
- Is the cheapest radionuclide?
- Is the most expensive radionuclide?
- Is the most dangerous?

THE SCIENCE OF RAD RATINGS

Scenarios (Game 2)

How to play:

- In groups of 3-6, shuffle the radionuclide cards and place the deck face down. Each player takes 3 cards but doesn't look at them yet.

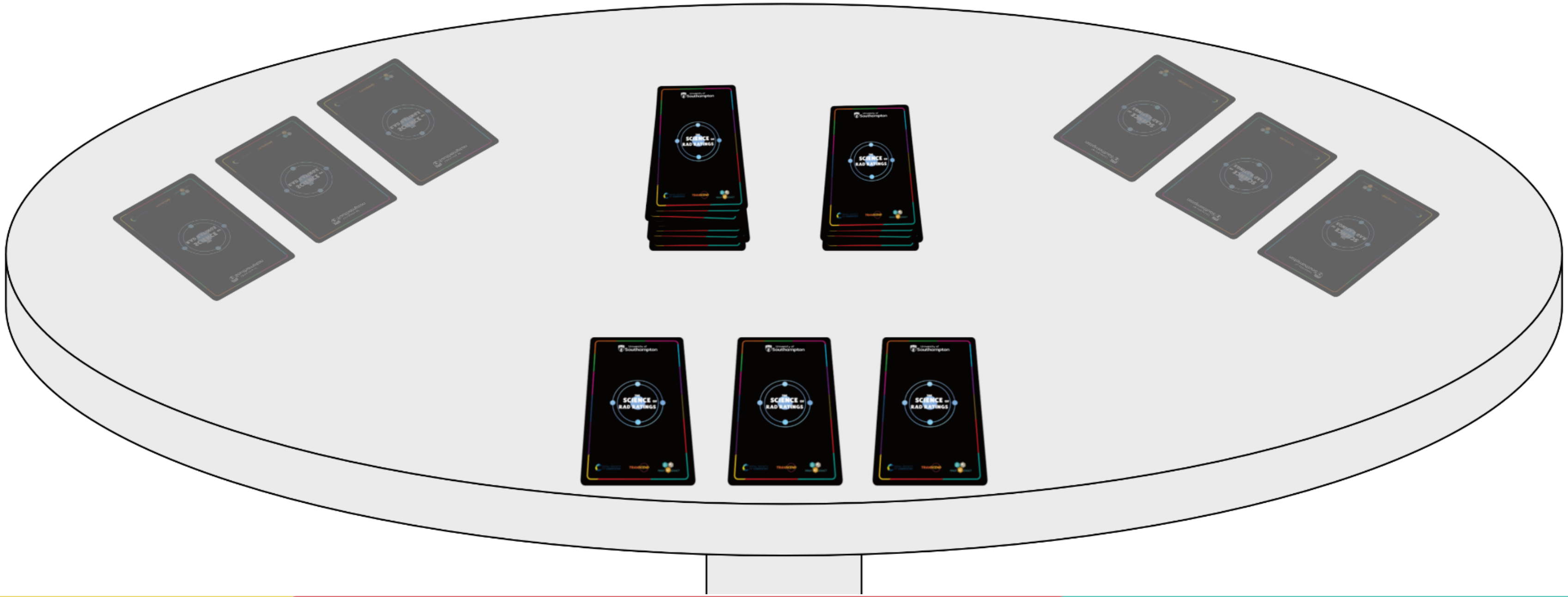

How to play:

- Player 1 chooses a scenario card and reads it aloud.
- Everyone looks at their own radionuclide cards and picks the isotope that they think is best suited for the job.

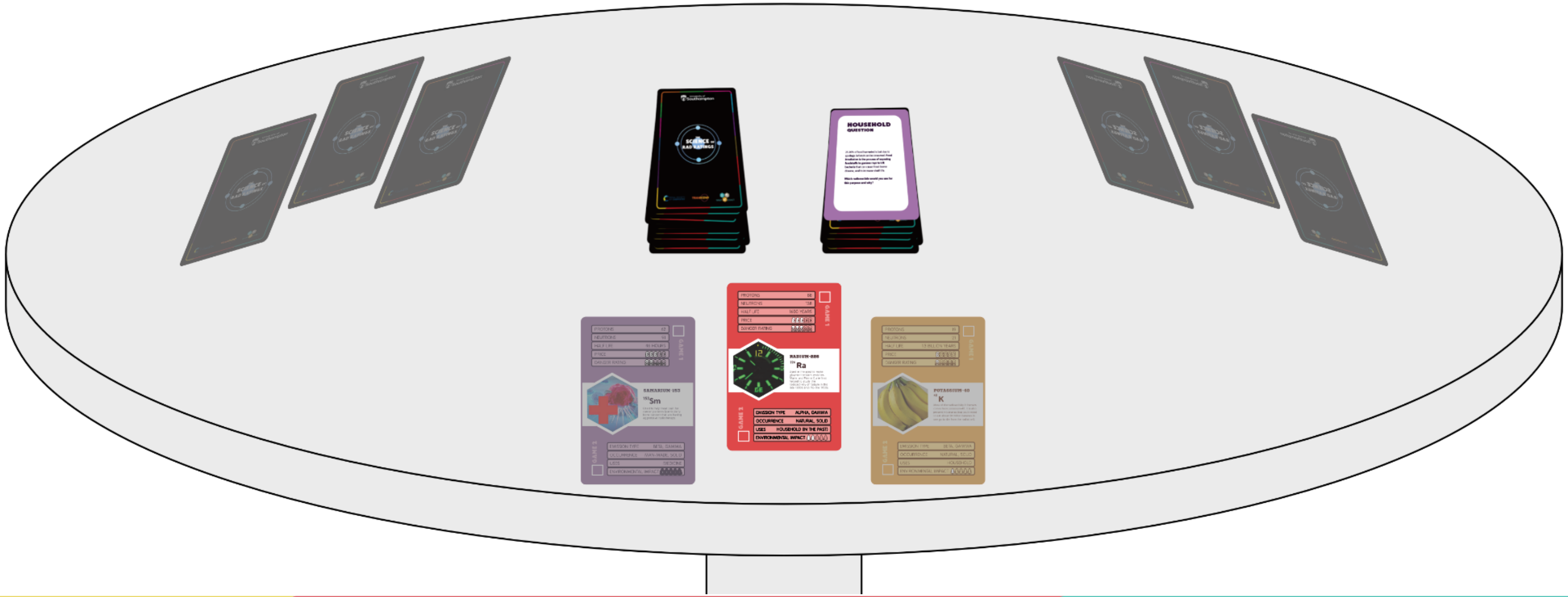

How to play:

- Each player presents their chosen card to the group and explains why they think it is the most appropriate radionuclide.
- The group votes on which card they think is best for the scenario - you cannot vote for yourself!

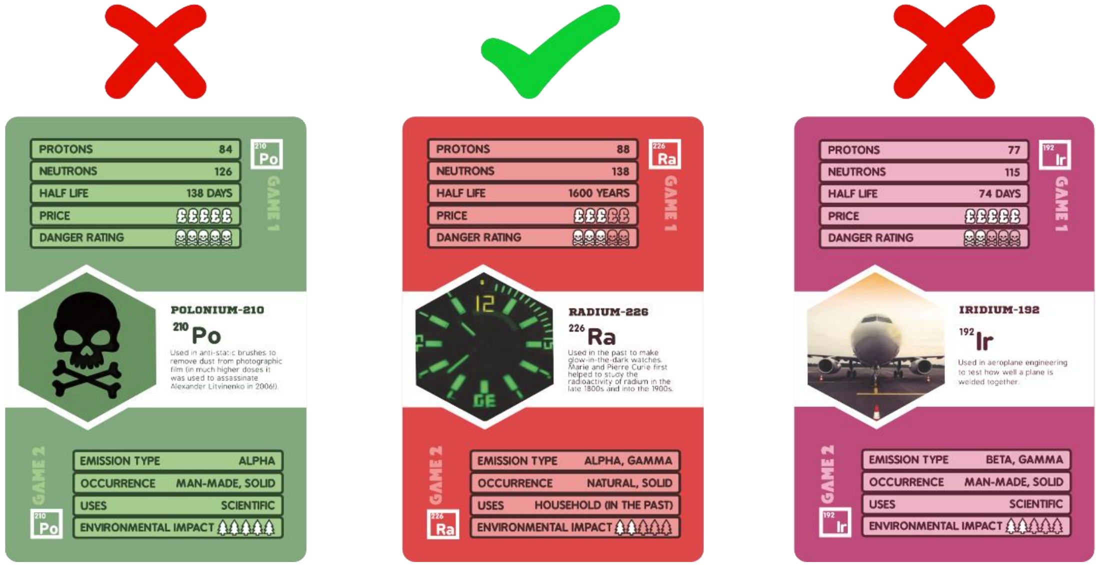

How to play:

- The card with the most votes gets put in that player's victory deck and all other cards are shuffled and returned to the bottom of the main deck. If there is a tie, both cards are put in their respective players’ victory decks.

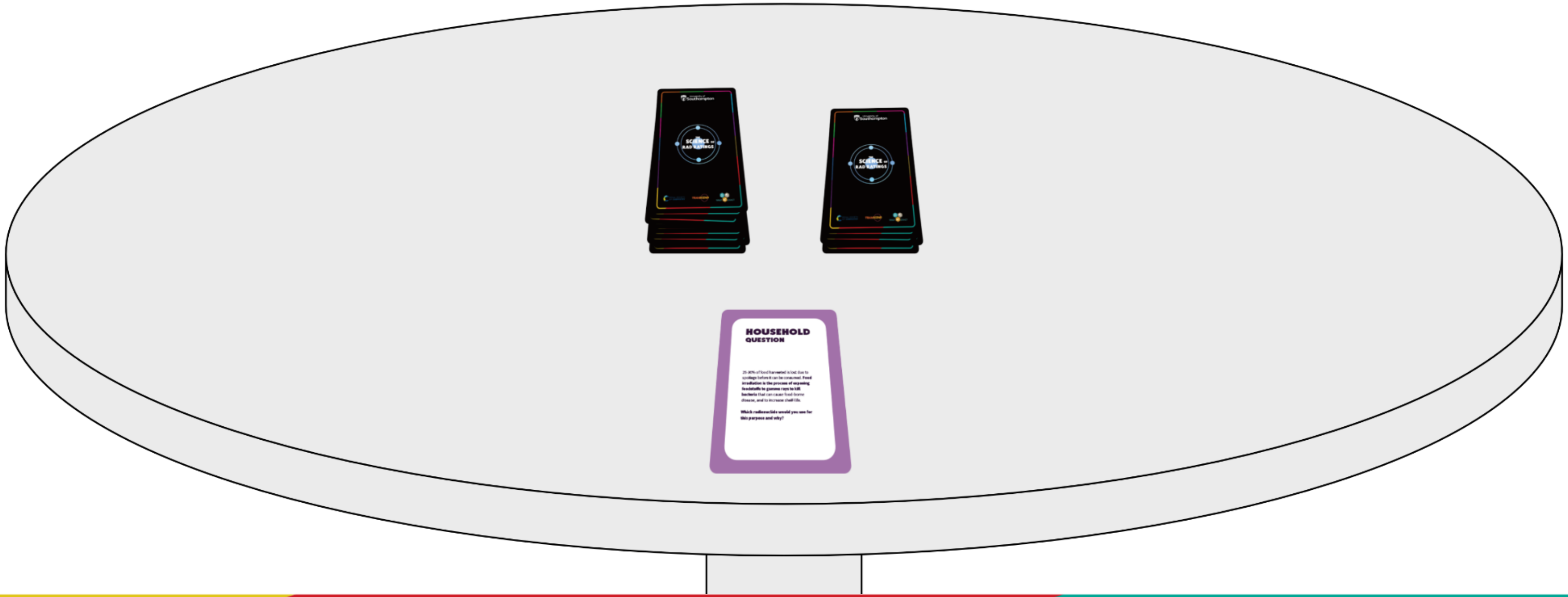

How to play:

- Player 2 picks the scenario for round 2, and so on.
- The first player to have 4 cards in their victory deck wins!

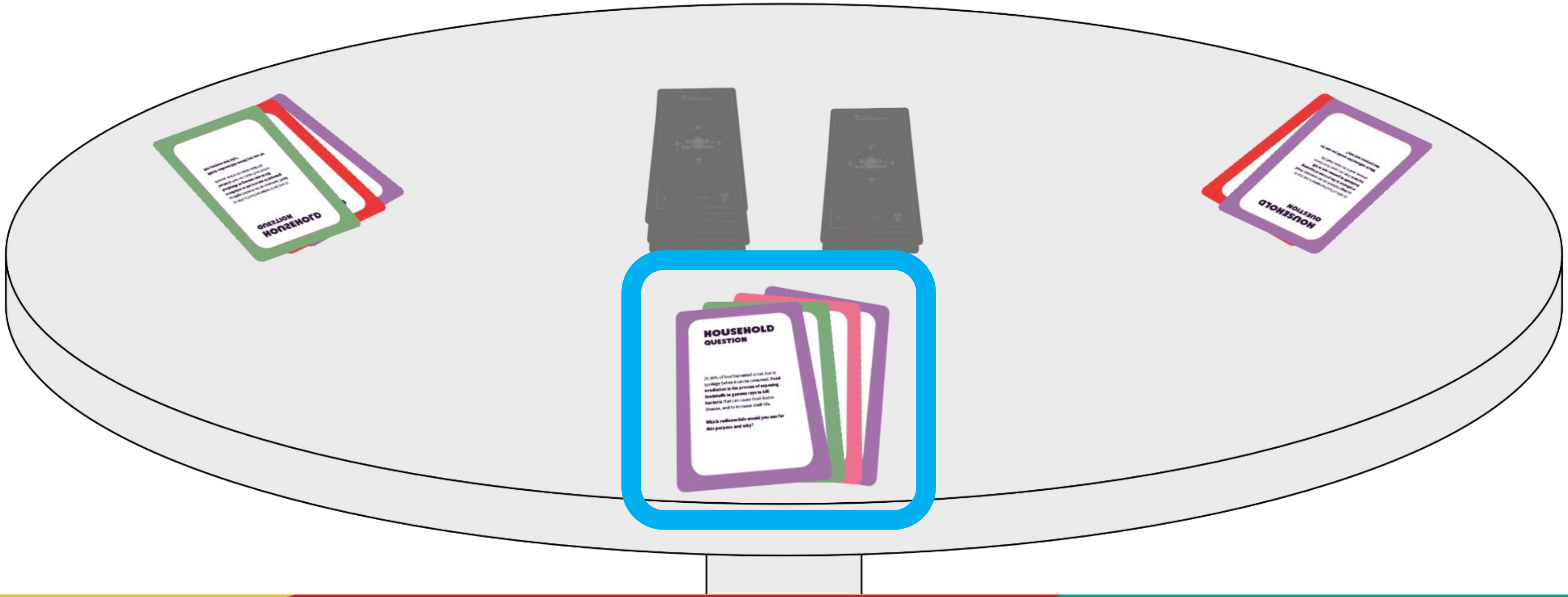

How to play Game 2 as a whole class:

- The class is separated into groups of 5 or 6. Each group gets a pack of radionuclide cards.
- Distribute the radionuclide cards evenly between each player in the group.
- Each player must pick the **most appropriate card** in their hand for the application described in the scenarios shown on the following slides.
- The answers will be shown after each question. **Remember to keep track of your score!**
- Each player can then return their card to their hand. **You can reuse this card in the next round.**
- The player with the most points at the end, wins!

How to play Game 2 as a whole class:

- The class is separated into groups of 5 or 6. Each group gets a pack of radionuclide cards.
- Distribute the radionuclide cards evenly between each player in the group.
- Each player must pick the **most appropriate card** in their hand for the application described in the scenarios shown on the following slides.
- The answers will be shown after each question. **Remember to keep track of your score!**
- Each player can then return their card to their hand. **You can reuse this card in the next round.**
- The player with the most points at the end, wins!

Household Questions

Smoke detectors work by a radionuclide emitting particles which ionise the air.

Which is the best radionuclide to use for this purpose and why?

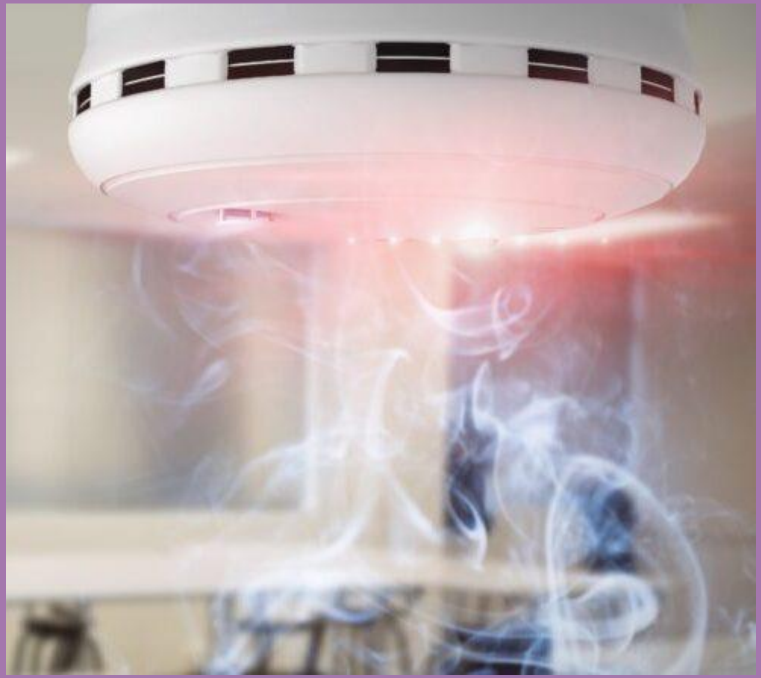

5 points

Americium-241

(alpha emitter, solid, suitable half-life; used in real life)

Radium-226

(alpha emitter, solid, suitable half-life)

3 points

Thorium-232

(alpha emitter, solid, but long half-life)

1 points

Uranium-238

(alpha emitter, solid, but long half-life)

Californium-252

(alpha emitter, solid, but short half-life and expensive)

Certain radionuclides release particles during decay, triggering a chemical reaction upon contact with other materials that creates a 'glow'. This reaction is known as **radioluminescence.**

You are travelling on a research expedition to the Arctic Circle in winter where it is dark 22 hours of the day. **You require a compass that can be read easily, which radionuclide would you use for this purpose and why?**

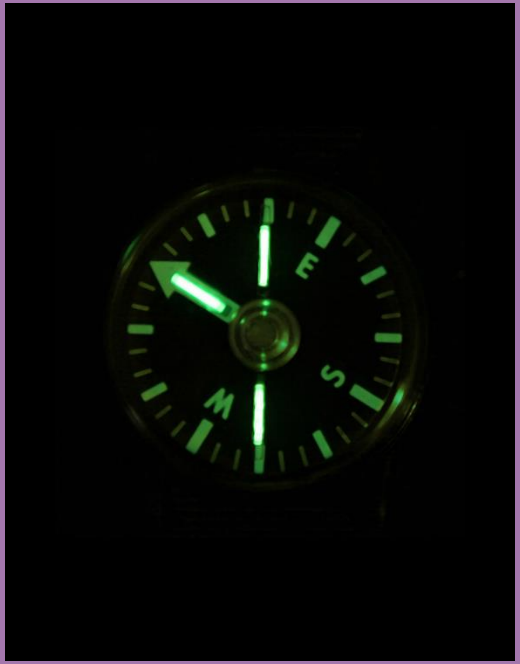

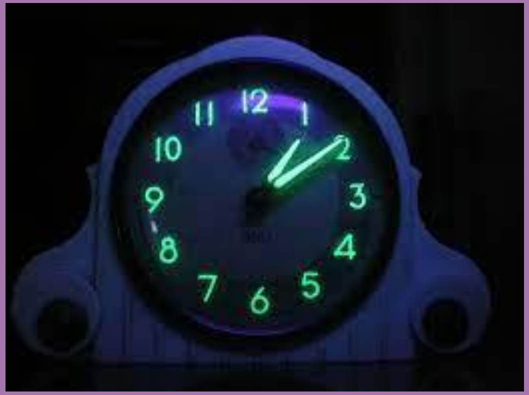

5 points

Tritium
(beta emitter, solid; used in real life)

Promethium-147
(beta emitter, solid; was used before tritium)

3 points

Radium-226
(alpha emitter, solid but long half-life + poisonous; was used before promethium-147)

Potassium-40
(beta emitter, solid, cheap, but long half-life)

1 points

Lead-210
(alpha emitter, solid, cheap, suitable half-life, but poisonous)

Nickel-63
(beta emitter, solid, but long half-life)

Medicine Questions

Diagnostic techniques in nuclear medicine use radiopharmaceuticals (or radiotracers) which emit particles or rays from within the body.

These tracers are generally short-lived isotopes, and their emissions are detected by the imaging device.

A patient needs to have the gas exchange in their lungs investigated, which radionuclide would you use for these investigations and why?

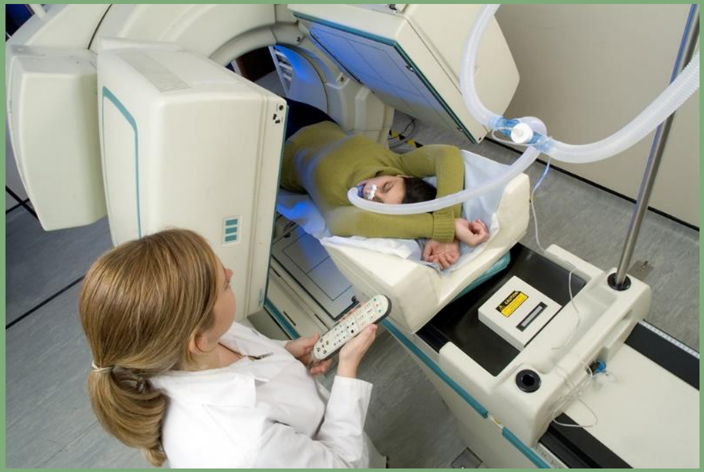

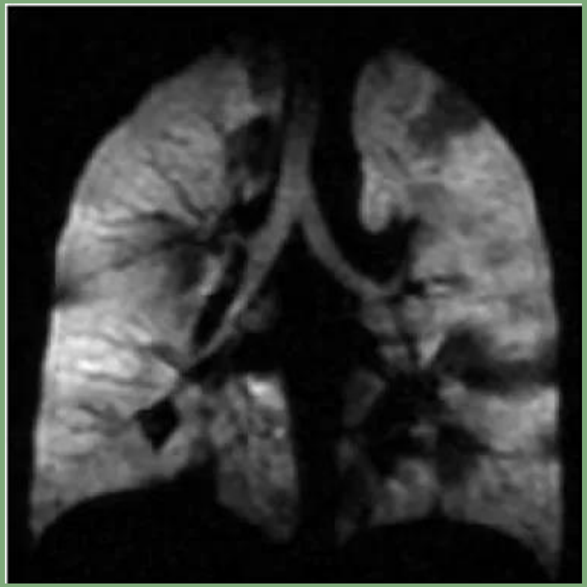

5 points

Xenon-133

(gamma emitter, gaseous (can be breathed in), suitable half-life; used in real life

3 points

Iodine-131

(gamma emitter, suitable half-life but solid (although it decays to xenon-131 which is a gas))

1 points

Technetium-99m

(gamma emitter, suitable half-life, but solid)

Nuclear medicine is also used for therapeutic purposes.

Which radionuclide would you use to treat a cancerous tumour affecting the thyroid gland?

Please explain your choice!

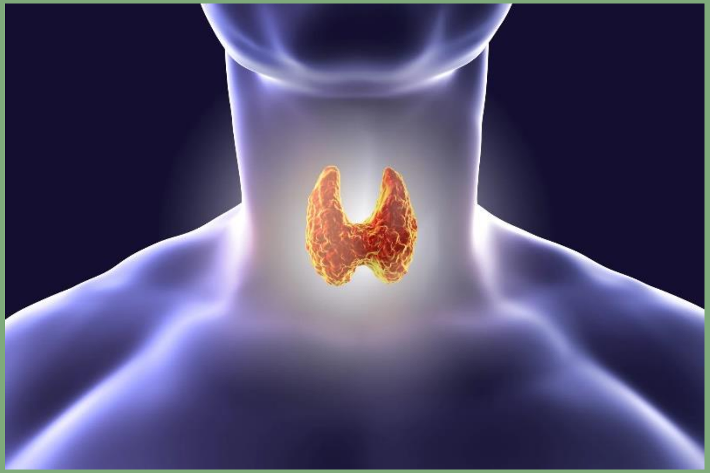

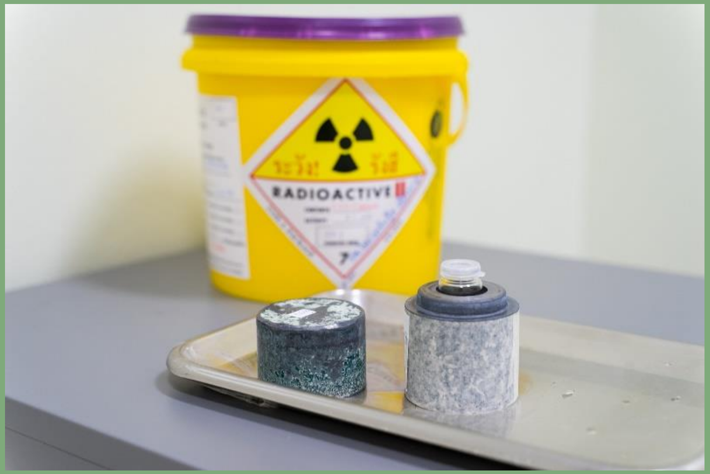

5 points

Iodine-131

(concentrates in the thyroid, solid, suitable half-life)

3 points

Iodine-123

(concentrates in the thyroid, solid, suitable half-life)

1 points

Nuclear Power Questions

Advanced Gas-cooled Reactors (AGRs) are the second generation of British gas-cooled reactors.

These reactors, like other nuclear technology, use the energy released by splitting atoms of certain elements.

Which is the best radionuclide to use as a fuel for an AGR and why?

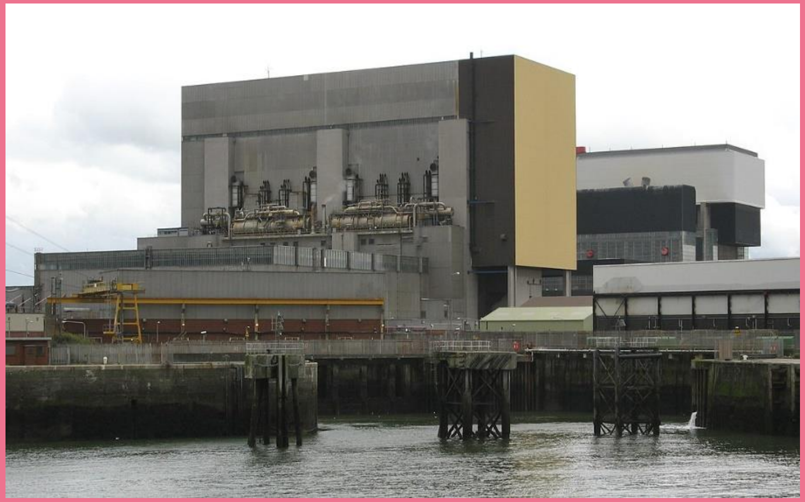

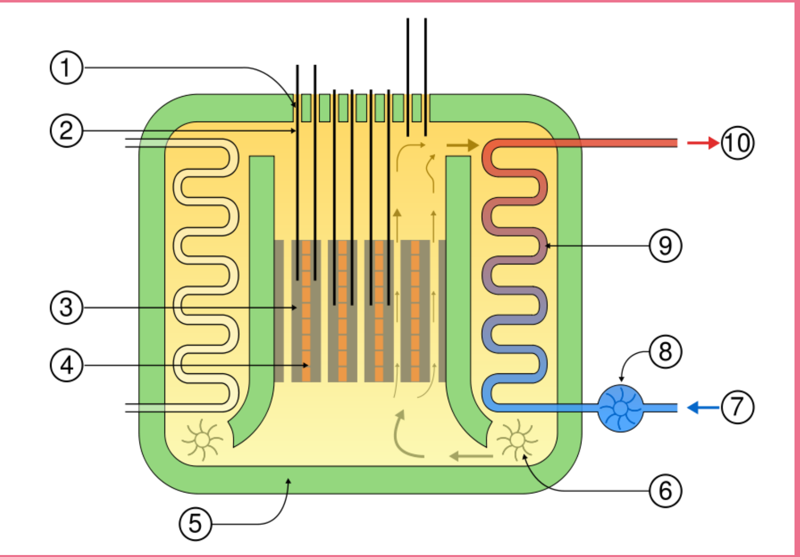

5 points

Thorium-232
(alpha emitter, solid, suitable half-life; used in real life)

Uranium-235
(alpha emitter, solid, suitable half-life; used in real life)

3 points

Plutonium-238
(alpha emitter, solid, suitable half-life, but expensive compared to thorium and uranium)

1 points

Uranium-238
(alpha emitter, solid, suitable half-life, but does not burn efficiently in nuclear reactors)

Americium-241
(alpha emitter, solid, suitable half-life but expensive compared to thorium and uranium)

Radioisotope Thermoelectric Generators (RTGs) have been used as an electricity source in spacecraft since 1961 **due to their high decay heat and short range of particle emissions.**

You are in charge of selecting an appropriate radionuclide to power a **50-year satellite Mars mission, which radionuclide do you choose to power your satellite and why?**

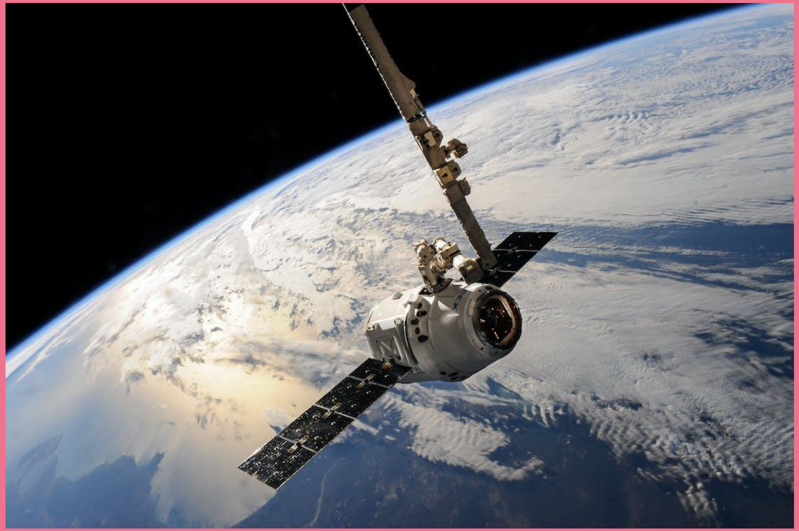

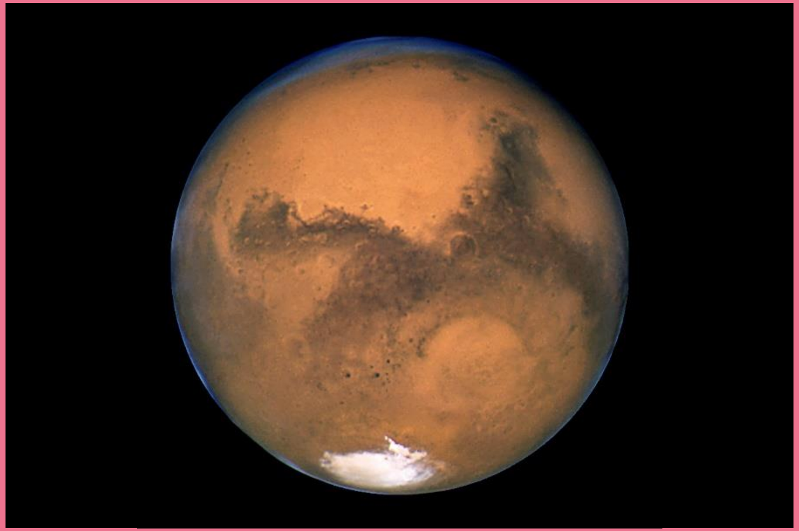

5 points

Plutonium-239

(alpha emitter, solid, suitable half-life; used in real life)

3 points

Americium-241

(alpha emitter, solid, but long half-life (so lots would be needed))

1 points

Science Questions

Efficient use of fertilisers is a concern to both developing and developed countries.

Fertilisers 'labelled' with a particular isotope provides a means of **finding out how much is taken up by the plant** and how much is lost into the environment.

Which radionuclide would you use for this purpose and why?

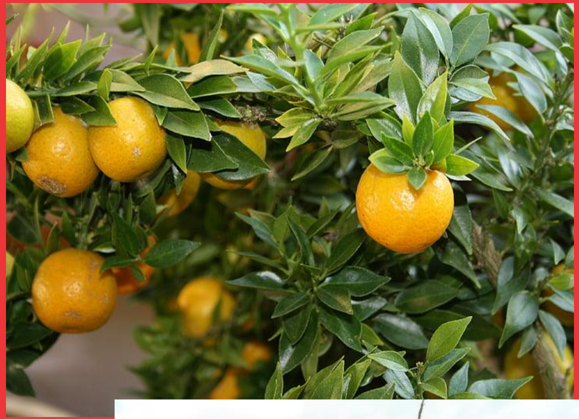

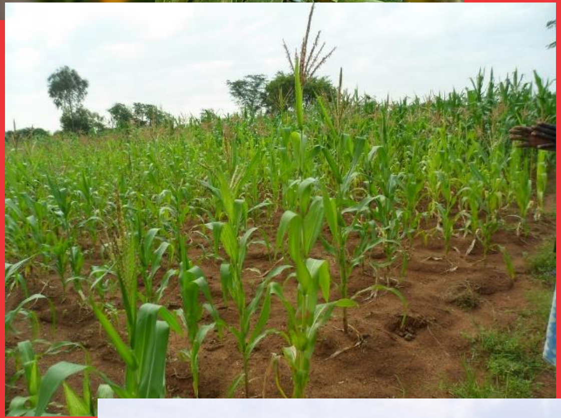

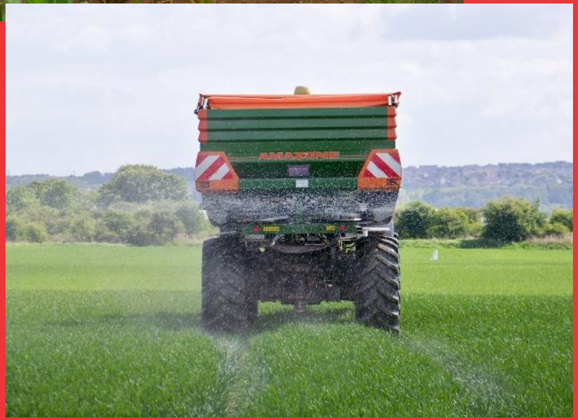

5 points

Phosphorus-32
(beta emitter, solid, suitable half-life, low environmental impact; stable phosphorus is an essential plant nutrient and so phosphorus-32 is used in real life)

3 points

Calcium-47
(beta + gamma emitter, solid, suitable half-life, low danger and environmental impact, stale calcium is a plant nutrient, but calcium-47 is man made)

1 points

Potassium-40
(beta + gamma emitter, solid, cheap, natural, low danger, low environmental impact, stable potassium is a plant nutrient, but potassium-40 has a very long half-life)

Analysing naturally-occurring radioisotopes is important for **determining the age of rocks and other materials** that are of interest to geologists, anthropologists, and archaeologists, among others.

A historian thinks the wooden beams in an old house were made in the early Tudor period (about **500 years ago**). **Which radionuclide would you use to check the age of the wood**, and therefore the age of the house?

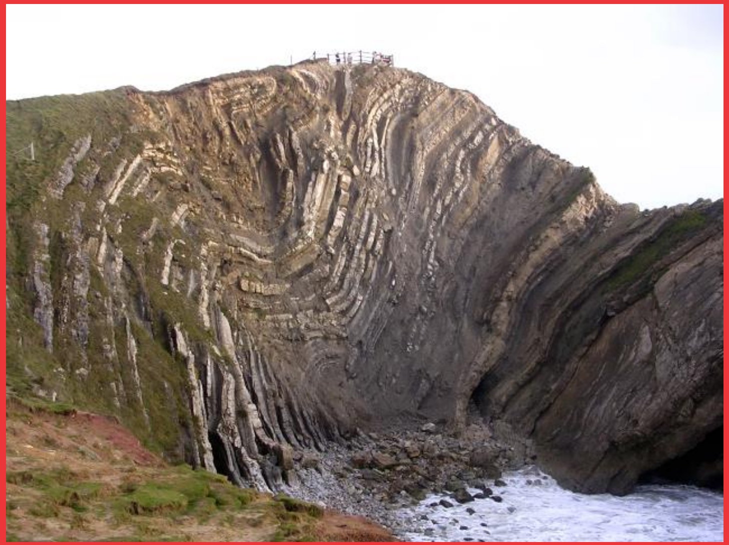

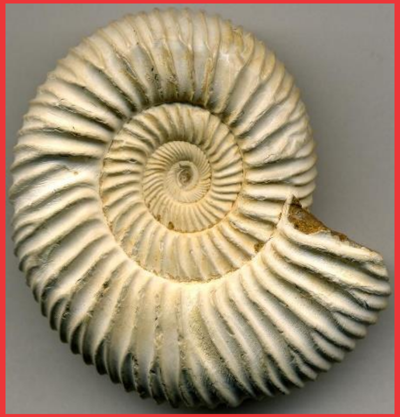

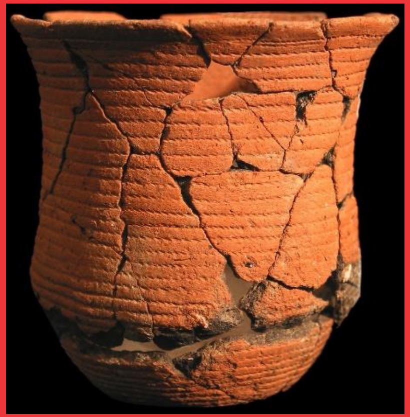

5 points

Carbon-14

(solid, suitable half-life, abundant in wood; used in real life)

3 points

Tritium

(solid, suitable half-life, found in wood although it is rare, so carbon-14 gives a more reliable age)

1 points

Caesium-137

(solid but not found in wood older than ~1950; better for dating sediments)

Lead-210

(solid but not found in wood and half life is too short; better for dating sediments)

Bonus Round!

Food irradiation is the process of exposing foodstuffs to gamma rays to kill bacteria that can cause food-borne disease, and to increase shelf-life.

Which radionuclide would you use for this purpose and why?

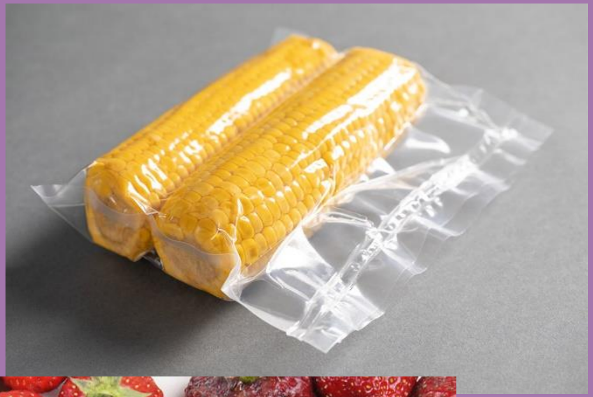

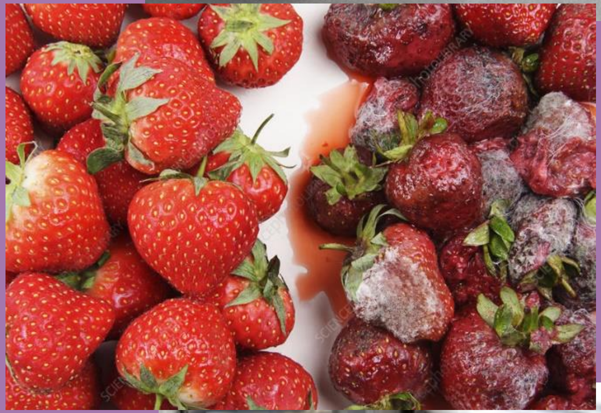

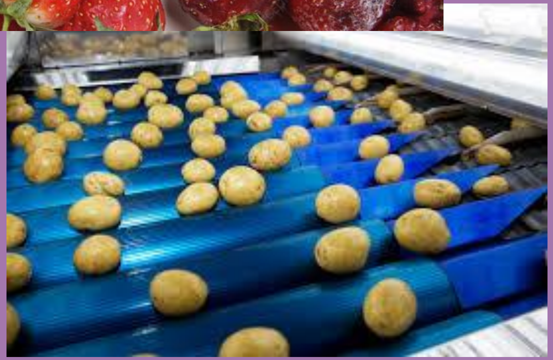

5 points

Cobalt-60
(gamma emitter, solid, suitable half-life; used in real life)

Caesium-137
(gamma emitter, solid, suitable half-life; used in real life)

3 points

Iron-55
(gamma emitter, solid, but short half-life)

Nickel-63
(gamma emitter, solid, but long half-life)

1 points

Iridium-192
(gamma emitter, solid, but expensive)

Promethium-147
(gamma emitter, solid, but expensive)

With any therapeutic procedure, the aim is to confine the radiation to well-defined target volumes of the patient.

There are several radionuclides that are used for the relief of cancer-induced bone pain.

Which radionuclides would you use to target cancerous growths in the bone and why?

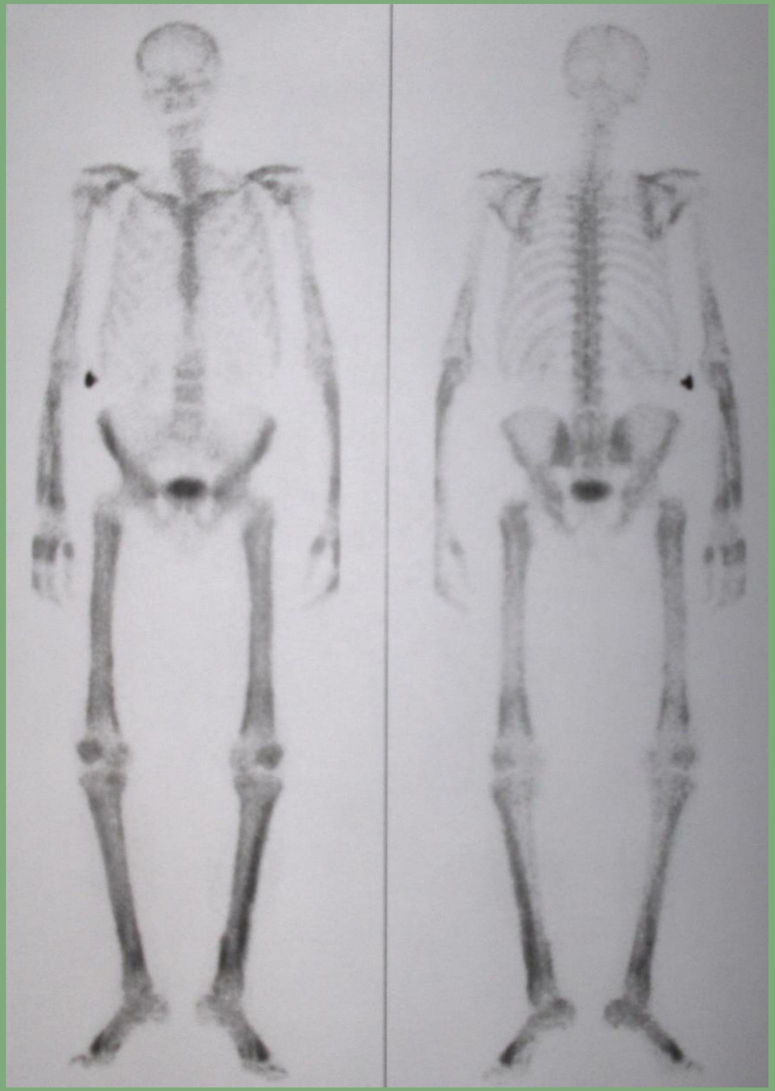

5 points

Calcium-47
(beta emitter, solid, suitable half-life, easily absorbed by bone; used in real life)

Samarium-153
(beta emitter, solid, suitable half-life)

3 points

Yttrium-90
(beta emitter, solid, easily absorbed by bone, but has a short half-life, better suited for treating liver cancer)

1 points

Strontium-90
(beta emitter, solid, cheap, easily absorbed by bone, but has a high environmental impact)

Caesium-137
(beta emitter, solid, but long half-life and not easily absorbed by bone)

Nuclear power is particularly suitable for vessels which need to be at sea for long periods without refuelling, or for powerful submarine propulsion.

Which radionuclide would you chose to power a submarine and why?

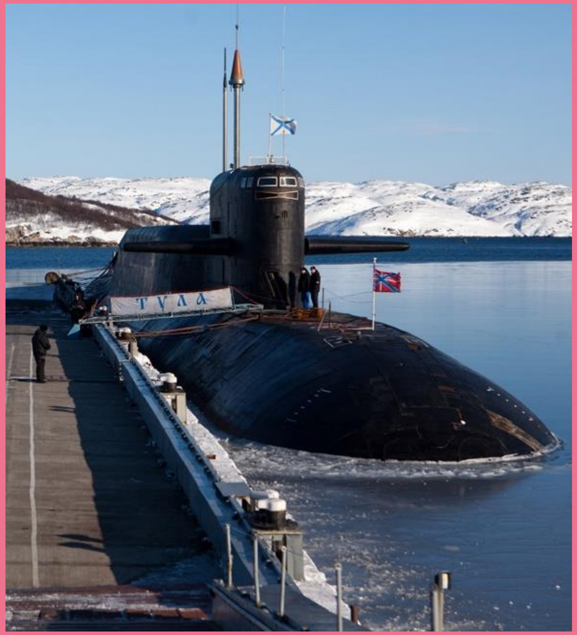

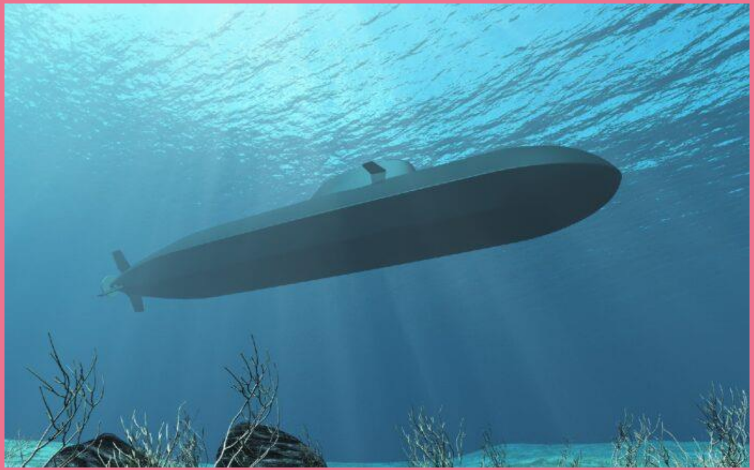

5 points

Thorium-232
(alpha emitter, solid, suitable half-life; used in real life)

Uranium-235
(alpha emitter, solid, suitable half-life; used in real life)

3 points

Plutonium-238
(alpha emitter, solid, suitable half-life, but expensive compared to Thorium and Uranium)

1 points

Uranium-238
(alpha emitter, solid, suitable half-life, but does not burn efficiently in nuclear reactors)

Americium-241
(alpha emitter, solid, suitable half-life but expensive compared to thorium and uranium)

Radioisotopes can be used to **accurately measure the thickness of many different sheet materials.**

They measure the amount of radiation from a source which has been absorbed in materials.

Which radionuclide would you use to **measure the thickness of an extruded metal pipe, why did you choose this radionuclide?**

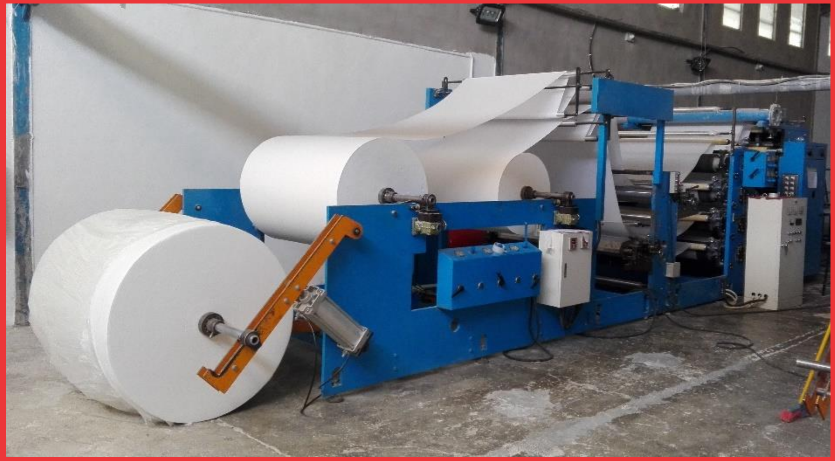

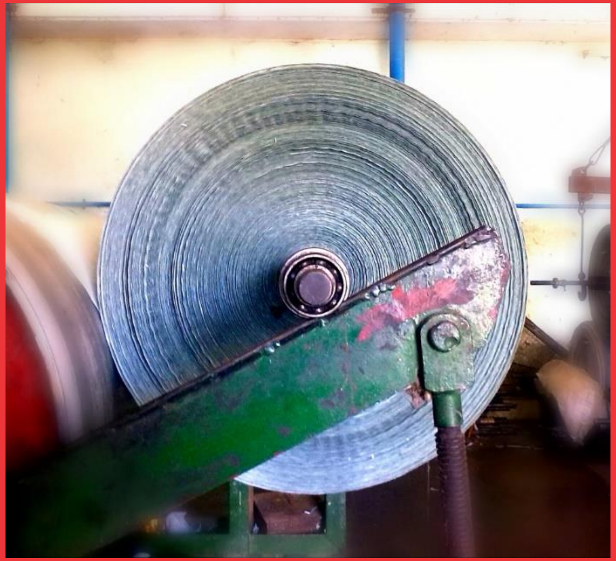

5 points

Iridium-192

(beta emitter, solid, suitable half-life; used in real life)

3 points

Cobalt-60

(beta emitter, solid, suitable half-life, cheap, but high danger rating)

Iron-55

(beta emitter, solid, suitable half-life, cheap)

1 points

Nickel-63

(beta emitter, solid, cheap, but long half-life)
