# Supplementary material for: Decked Out for Success: A Novel Card Game to Support School Teaching of Radioactivity and Nuclear Science
Source: J Chem Educ. 2024 Dec 18;102(1):430–6. doi: 10.1021/acs.jchemed.4c00603 (PMC11736787; doi:10.1021/acs.jchemed.4c00603)
Supplement: Supplementary file 3 — ed4c00603_si_003.pdf [file ed4c00603_si_003.pdf]

Supplementary information 3

Decked out for success: a novel card game to support school teaching of radioactivity and nuclear science

Sarah E. Lu[1*], Shaun D. Hemming[1], Jamie M. Purkis[1]
[1]University of Southampton, University Road, Southampton SO17 1BJ, United Kingdom (UK)
*Corresponding author: Sarah Lu, Sarah.Lu@soton.ac.uk

Contents:
Creation of the cards
Game distribution

Creation of the cards

Conceptualization and Planning: The initial phase included brainstorming and defining the game's purpose, objectives, and educational goals. Multiple exam-board syllabi for ages 14-18 were examined to identify the extent of radioactivity and radiochemistry taught at schools, as well as the ages at which students encounter the material. This analysis guided the development process to ensure the game effectively supplemented the existing curriculum, with a specific focus on engaging KS4 (age 14-16) students.

Educational Content Design: With the educational objectives in mind, the game's primary content was designed. This involved the creation of 30 unique and brightly coloured, distinctive cards to demonstrate the wide array of applications within nuclear and various STEM industries, as well as sectors including nuclear medicine. Feedback from students who had played Isotope Rummy stated that fun facts and pictures would have improved the game and their grasp on applications of radionuclides, so these were incorporated to each radionuclide card from the start of the education design.[20] Radionuclide content covered 10 named radionuclides in the English KS4 syllabus as well as examinable curriculum topics such as physical properties of nuclear structure, half-life, radiation, products of radioactive decay, risk assessment, and applications. The game layout and design also underwent evaluation by a accessibility practitioner to ensure cards were readable from arm's length and the color palette utilized accessible combinations with accessible color palette builder to ensure a color contrast ratio of 4:5:1 was met.

Game Mechanics and Rules: Gameplay mechanics and rules were then developed to ensure the game was both educational and enjoyable. This was done by beta testing groups with postgraduate students and oral feedback. This included determining how players would interact with the cards and other players, establishing win conditions, and structuring progression in the game.

Playtesting and Iteration: To refine the game, the team conducted extensive playtesting sessions. This involved observing how players interacted with the game, identifying any potential issues or areas for improvement, and gathering feedback from participants. This iterative process was crucial in fine-tuning the game's mechanics and ensuring its effectiveness as a fun and educational tool.

Integration with Educational Objectives: The game was designed to align and complement educational objectives of the KS4 curricula. It aimed to reinforce key concepts in radioactivity and radiochemistry while emphasizing the practical applications of these subjects in real-world scenarios to highlight potential career paths.

Collaborative Partnerships: The development process benefitted from collaborative partnerships with the teachers, outreach and engagement specialists, educational accessibility practitioners as well as the Royal Society of Chemistry, the TRANSCEND Consortium. These partnerships provided additional expertise and perspectives throughout the project, contributing to the game's educational accessibility and real-world applicability.

Creation of Supporting Materials: In addition to the physical cards, supporting materials such as a PowerPoint presentation to enhance the educational experience were developed in conjunction with teacher feedback. These materials were designed to complement and reinforce the lessons taught through gameplay.

Overall, the development of the *RAD Ratings* card game was a comprehensive and collaborative effort, leveraging the expertise and resources of the University of Southampton, along with its partners, to create an engaging and educational tool for teaching radioactivity and radiochemistry to KS4 students. Through careful planning, iterative design, and alignment with educational objectives, the team successfully produced a valuable resource for both teachers and students.

GAME DISTRIBUTION

Upon completion of the pilot stage, *RAD Ratings* was made available to request without cost for schools, universities, and industrial STEM ambassadors. The distribution between these groups is shown in Table 1. This was achieved by a Microsoft form where the requestor would enter the sector that best described their workplace. Feedback from STEM organizers involved in school engagement activities highlights the significant positive impact of utilizing pre-prepared card games. Specifically, utilizing of pre-prepared card games and learning materials substantially streamlined the preparation process, resulted in a notable reduction in time commitments for school visits and engagements. Moreover, STEM ambassador feedback highlighted those pre-prepared materials effectively lowered participation barriers, allowing for more engagement interactions with schools. This observation highlights the practical advantages of incorporating gamified educational tools in outreach initiatives, ultimately enhancing the effectiveness and efficiency of these valuable educational endeavors.

**Workplace/Sector**	**Proportion of Cards Distributed**
Universities/ Academia	16%
Government/ Public Sector	8%
Private Sector	26%
Schools	50%

Table S1. Percentage of cards distributed to different sectors.
